# Elevated pre-supplementary motor area activity during reward expectancy: An impulsivity-related neural marker of vulnerability to bipolar and externalizing disorders

**DOI:** 10.1038/s41380-026-03528-0

**Published:** 2026-03-19

**Authors:** Robert Raeder, Manan Arora, Michele Bertocci, Henry W. Chase, Alexander S. Skeba, Genna Bebko, Haris A. Aslam, Simona Graur, Osasumwen Benjamin, Yiming Wang, Richelle Stiffler, Mary L. Phillips

**Affiliations:** Department of Psychiatry, Western Psychiatric Institute and Clinic, University of Pittsburgh Medical Center, University of Pittsburgh, Pittsburgh, PA USA

**Keywords:** Neuroscience, Psychology

## Abstract

Mania/hypomania is pathognomonic of bipolar disorder (BD), yet early identification remains challenging. Impulsivity is a key feature of mania/hypomania and of externalizing disorders that may predispose to BD, but neural markers of impulsivity-related risk remain unknown. This study aimed to identify reward expectancy (RE)-related neural correlates of impulsivity facets, test moderation by current affective/anxiety symptoms, and determine whether such markers differentiate BD and/or externalizing disorders from low impulsivity individuals. Two independent BD-risk samples aged 18–30 years, including individuals with prior externalizing disorder diagnoses but not BD, were recruited; a euthymic BD group was also recruited. Impulsivity facets were assessed via Behavioral Activation System (BAS) and UPPS-P scales. Whole-brain regressions identified neural correlates of impulsivity facets during RE. Linear models tested replication and current affective/anxiety symptom moderation. ANCOVA compared neural activity among BD, externalizing, and non-BD/externalizing impulsivity tertile groups. Whole-brain regressions revealed a positive association between BAS Fun Seeking and pre-supplementary motor area (pre-SMA) activity (*p*_FWE_ = 0.003, *k* = 167), which replicated when depressive symptoms were covaried (discovery: *β* = 2.73, *p* < 0.001; replication: *β* = 0.88, *p* = 0.036; combined: *β* = 1.49, *p* < 0.001). A significant pre-SMA × depression interaction (*β* = –0.08, *p* = 0.037) indicated depressive symptoms attenuated the pre-SMA-Fun Seeking association. Group comparisons revealed greater pre-SMA activity in high-Fun Seeking (*p* < 0.001) and externalizing disorder groups (*p* = 0.039) versus low-Fun Seeking, with similar trends observed in BD once individuals taking medications, particularly benzodiazepines (*p* = 0.012), were excluded. Pre-SMA hyperactivity during RE is a robust neural correlate of BAS Fun Seeking, moderated by depression severity. This pattern represents a trait-linked neural marker of impulsivity associated with vulnerability to BD and externalizing disorders, informing early risk identification and intervention.

## Introduction

Bipolar disorder (BD) is a prevalent affective illness involving recurrent episodes of mania (BD type I, BDI) or hypomania (BD type II, BDII), alongside alternating or mixed depressive episodes [1, 2]. Accurately diagnosing BD is challenging, as depressive symptoms often resemble major depressive disorder (MDD), while manic/hypomanic symptoms are frequently underreported [3]. Given the deleterious consequences of misdiagnosis [4], and the importance of early identification [5], developing objective markers of mania/hypomania is a clinical priority.

Elevated impulsivity is a hallmark of mania/hypomania in BD, characterized by poorly regulated, rapid, risky, or inappropriate behaviors enacted with minimal forethought [1, 6–8]. Although more pronounced during manic/hypomanic episodes, impulsivity persists as a trait-level vulnerability [9], evident in euthymic [10] and remitted states [11], contributing to many adverse outcomes in BD. Impulsivity in BD is associated with increased symptom severity [12], impaired psychosocial functioning [13], increased risk for addiction-related comorbidities [14], higher incarceration rates [15], and heightened risk of suicidal behavior [16]—underscoring the need to elucidate its neurobiological underpinnings. Moreover, other externalizing disorders characterized by elevated impulsivity, such as attention-deficit/hyperactivity disorder (ADHD), substance-use disorders (SUD), or antisocial behaviors, often precede BD onset, or are comorbid with BD, and may serve as early clinical precursors of illness risk [17–19]. Understanding the neural basis of impulsivity in populations at risk for BD therefore has dual relevance: it elucidates neural mechanisms underlying BD vulnerability while also informing neural mechanisms underlying vulnerability to the broader range of externalizing disorders.

Impulsivity is not a unitary construct, but rather, comprises distinct cognitive and affective facets [20–22], each of which may differentially contribute to risk for BD or externalizing disorders. Importantly, contemporary models also emphasize that impulsivity reflects multiple partially overlapping neural mechanisms, including those underlying the trait-level facets of inhibitory control deficits, rash action, reward sensitivity, temporal discounting, and affect-driven urgency, rather than a single unitary disposition [23]. Several scales are therefore commonly used to assess impulsivity facets; among them, the Behavioral Activation System (BAS) [21] and Urgency, Premeditation, Perseverance, and Sensation Seeking–Positive Urgency (UPPS-P) [22] scales capture a broad range of these dimensions. As such, the neural mechanisms of impulsivity facets underlying vulnerability to BD and other externalizing disorders are likely to be complex and context dependent.

Reward expectancy (RE)—the anticipation of uncertain future reward—is a salient context for examining neural mechanisms underlying vulnerability to BD and other externalizing disorders, as it reliably engages prefrontal cortical-ventral striatal circuits implicated in dopamine-modulated motivation, reward processing, and approach behavior [24–32]. Prior work identified elevated left ventrolateral prefrontal cortex (L-vlPFC) activity during RE as a robust neural marker of mania/hypomania risk [33–35], as well as being observed in euthymic individuals with BD [36, 37]. This likely reflects both the broader role of the left prefrontal cortex in approach-related affective processing [38–40], as well as the specific involvement of the vlPFC in encoding the expected value of anticipated rewards [41, 42]. L-vlPFC activity during RE has also been shown to be positively associated with specific impulsivity facets [34, 43]. Other BAS and UPPS-P impulsivity facets have been associated with partially dissociable prefrontal patterns: emotion-driven urgency with reduced dorsolateral prefrontal and anterior cingulate engagement but heightened orbitofrontal–ventromedial prefrontal reactivity; and deficits in planning and control with lower dorsolateral prefrontal and anterior cingulate activity [44–47]. Notably, affective state, particularly depression, can modulate the expression of impulsivity: for example, during depressive episodes, ventral striatal activity is blunted and orbitofrontal-prefrontal cortical activity is elevated, in association with suppression of reward-driven approach behaviors, thereby dampening the outward expression of impulsivity despite elevated underlying vulnerability [48–50]. Consistent with this, studies directly comparing BD and MDD indicate that depressive state is associated with blunted ventral striatal and altered prefrontal responses during reward processing, highlighting both diagnosis- and state-dependent modulation of reward circuitry [51].

Despite these advances in elucidating neural correlates of some impulsivity facets, there is limited understanding of the neural mechanisms and associated neural markers underlying the broader range of impulsivity facets, and thus vulnerability to externalizing disorders and BD. As affective states modulate how trait-level impulsivity facets are expressed [48], a critical first step in identifying neural markers of vulnerability to the broader range of externalizing disorders is to identify neural markers of these impulsivity facets that are independent of the influence of affective symptoms, particularly depression [49, 50]. This approach will determine the extent to which identified neural markers of impulsivity facets do in fact reflect trait-level vulnerability markers, transient effects influenced by present affective state, or interactions among these factors.

Given the need for reproducibility in psychiatric neuroimaging [52], the present study leveraged two independent samples of young adults recruited across a range of vulnerability to future BD, including individuals with a lifetime presence of externalizing disorders, as well as a sample of euthymic individuals with BD, to: (1) identify neural markers of BAS and UPPS-P impulsivity facets during RE in the discovery at-risk sample; (2) test replication in an independent at-risk sample and then confirm in the combined at-risk sample; (3) evaluate whether current affective and anxiety symptoms influence or interact with these neural markers, potentially moderating the strength or direction of associations with impulsivity facets; and (4) contextualize the relative magnitude of these neural markers by comparing at-risk individuals, individuals with non-BD externalizing disorders, which can predispose to BD, and euthymic individuals with BD. We hypothesized that: (1) replicable neural markers of impulsivity facets would be identified during RE, localizing primarily to prefrontal cortical regions; (2) affective symptoms, especially depression severity, would moderate associations among these neural markers and impulsivity facets; and (3) that these neural markers would be of similar magnitude in at-risk individuals with high levels of impulsivity, as those with externalizing disorders, and those with BD.

## Materials and methods

Two independent at-risk samples were enrolled in a cross-sectional study examining neural markers of future BD risk between 2014 and 2023. Individuals aged 18–30 years were recruited from the Pittsburgh community via advertisements, participant registries, and local psychiatric services. All diagnostic assessments were conducted using the Structured Clinical Interview for DSM-5 Research Version (SCID-5-RV) [53]. The at-risk samples included individuals with subsyndromal-syndromal psychopathology related to externalizing disorders (e.g., ADHD or SUD) that can precede BD, but excluded individuals with BD. These samples also included individuals with subsyndromal-syndromal depressive and anxiety disorders, allowing assessment of potential moderating effects of depressive and anxiety symptoms on associations among neural activity during RE and impulsivity facets predisposing to externalizing disorders. Euthymic individuals with BD, including both BDI and BDII, given evidence of shared neurobiological mechanisms [37, 54, 55], were also recruited. All participants were fluent in English and provided informed consent. The study was approved by the University of Pittsburgh Human Research Protection Office.

Building on prior analyses [33], the present study expanded the discovery sample from this previous study, which was recruited across a full-range of risk for BD, to include newly recruited participants. The replication sample primarily comprised distressed, treatment-seeking individuals characterized by higher levels of state affective symptoms, providing the opportunity to test generalizability under conditions of heightened affective pathology. Impulsivity facets were assessed via BAS (assessing Drive, Fun Seeking, Reward Responsiveness) and UPPS-P (assessing Negative Urgency, Lack of Premeditation, Lack of Perseverance, Sensation Seeking, Positive Urgency) scales. The Hamilton Rating Scale for Depression (HRSD) assessed depression severity [56], the Hamilton Anxiety Rating Scale (HAMA) assessed anxiety severity [57], and the Young Mania Rating Scale (YMRS) assessed mania/hypomania severity [58]. Full methodological details are provided in the Supplementary Materials.

Participants completed a validated 16-minute event-related monetary card-guessing task designed to probe RE contexts during the anticipation of potential future reward, as outlined in prior reports [33, 34]. Each trial comprised a choice phase, an anticipation phase (2–6 s, jittered) during which cues signaled the potential for monetary reward or loss, followed by feedback and outcome. RE context in this task therefore reflects anticipatory motivational and approach-related processes under uncertainty. Neural activity during the anticipation phase was modeled using a parametric regressor reflecting expected value. Functional MRI data were acquired on a 3.0 Tesla Siemens MRI scanner and preprocessed using established pipelines. Motion-related artifacts were addressed through a combination of stringent exclusion criteria and denoising: participants exhibiting excessive head motion (mean framewise displacement >0.5 mm or maximum >5 mm) were excluded, and multi-echo independent component analysis was applied to reduce residual motion- and physiology-related noise. Full details of task design, image acquisition, preprocessing, and quality-control procedures are provided in the Supplementary Materials.

The discovery sample included 143 participants; one outlier was removed via Z-score thresholding following initial whole-brain regressions (see methods below), yielding a final sample of 142. Replication was tested in an independent sample of 122 participants. The euthymic BD sample included 37 individuals. Sample characteristics are provided in Table 1.

**Table 1:** Characteristics for all samples.

Characteristic	Non-BD Samples		Euthymic BD Sample
	Discovery Sample	Replication Sample	
Sample Size, N	143	122	37
**Sex**			
Female, n(%)	96 (67.1%)	89 (73.0%)	30 (81.1%)
Male, n(%)	46 (32.2%)	33 (27.0%)	7 (18.9%)
Age, mean(SD)	23.79 (3.32)	21.67 (2.12)	25.13 (3.81)
NART IQ, mean(SD)	110.04 (7.40)	108.36 (7.50)	112.22 (5.89)
Current diagnosis of BD I, n(%)	NA	NA	12 (32.4%)
Current diagnosis of BD II, n(%)	NA	NA	25 (67.6%)
History of any depressive disorder, n(%)	27 (19%)	89 (73%)	1 (2.7%)
History of any anxiety disorder, n(%)	20 (14.1%)	79 (64.8%)	23 (62.2%)
History of any externalizing disorder (including ADHD), n(%)	15 (10.6%)	43 (35.2%)	25 (67.6%)
History of ADHD (specific), n(%)	7 (4.9%)	12 (9.8%)	18 (48.6%)
History of any trauma-related disorder, n(%)	10 (7%)	20 (16.4%)	6 (16.2%)
History of any substance-use disorder, n(%)	8 (5.6%)	19 (15.6%)	12 (32.4%)
History of any OCD-related disorder, n(%)	5 (3.5%)	17 (13.9%)	4 (10.8%)
History of any eating disorder, n(%)	7 (4.9%)	6 (4.9%)	6 (16.2%)
**BAS Scores**			
Drive, mean(SD)	11.06 (2.25)	11.29 (2.76)	11.43 (2.24)
Fun Seeking, mean(SD)	11.59 (2.30)	11.82 (2.43)	13.00 (2.11)
Reward Responsiveness, mean(SD)	17.06 (2.17)	16.99 (2.33)	18.00 (1.78)
BAS Activation Total, mean(SD)	39.70 (5.44)	40.10 (6.20)	42.43 (4.59)
**UPPS-P Scores**			
Negative Urgency, mean(SD)	2.05 (0.65)	2.56 (0.58)	2.94 (0.60)
(Lack of) Premeditation, mean(SD)	1.79 (0.45)	1.90 (0.57)	2.17 (0.55)
(Lack of) Perseverance, mean(SD)	1.91 (0.51)	2.24 (0.50)	2.42 (0.51)
Sensation Seeking, mean(SD)	2.79 (0.67)	2.70 (0.63)	2.79 (0.68)
Positive Urgency, mean(SD)	1.59 (0.59)	1.87 (0.68)	2.51 (0.74)
UPPS-P Total, mean(SD)	2.02 (0.42)	2.25 (0.42)	2.57 (0.46)
YMRS Score, mean(SD)	0.40 (0.92)	2.67 (1.86)	1.16 (1.38)
HRSD Score, mean(SD)	2.15 (3.76)	14.79 (6.25)	5.86 (4.47)
HAMA Score, mean(SD)	1.70 (3.10)	12.13 (5.96)	4.14 (3.01)
Taking psychotropic medication, n(%)	1 (0.7%) Antidepressant (1)	6 (4.9%) Antidepressant (4); Mood Stabilizer (2)	37 (100%) Antidepressant (14); Benzodiazepine (2); Mood Stabilizer (26); Antipsychotic (11)
Duration of BD Illness, mean(SD)	NA	NA	9.64 (4.69)
Total Number of Mood Episodes, mean(SD)	NA	NA	6.14 (4.90)
Number of Manic/hypomanic Episodes, mean(SD)	NA	NA	2.50 (2.01)
Number of Depressive Episodes, mean(SD)	NA	NA	4.53 (4.12)

### Aim 1: Identify neural correlates of impulsivity facets in the discovery sample

As the neural correlates of approach-related impulsivity are not fully established, a whole-brain analytic approach was implemented. Given moderate-to-high correlations among BAS and UPPS-P impulsivity facets, separate whole-brain regressions were conducted using Statistical Parametric Mapping, Version 12 (SPM12; Wellcome Centre for Human Neuroimaging) to identify unique associations between each impulsivity facet and neural activity during RE. Intercorrelations among BAS and UPPS-P impulsivity facets are reported in Supplementary Materials. Although separate modeling did not control for shared variance, it reduced multicollinearity and improved interpretability of facet-specific correlates. Each model tested one impulsivity facet as the primary predictor, assessing both positive and negative contrasts, controlling for age, sex, and IQ. Whole-brain models were thresholded at voxel-wise *p* < 0.001 (uncorrected), with statistical significance determined by comparison of cluster-level family-wise error (FWE) values to a Bonferroni-adjusted threshold (*p*_FWE_ = 0.006; 0.05/8) to account for eight impulsivity facet models. This cluster-level correction controlled for Type I errors [59, 60]. To ensure findings were not confounded by cognitive ability, sensitivity analyses excluded IQ to assess its influence on observed effects, given its relevance to response inhibition and impulsivity [61].

### Aim 2: Replication via extracted beta values

To test replication, masks of significant clusters identified in the discovery sample were applied to the replication sample in SPM12 to extract beta values of neural activity during RE. Beta values were similarly extracted in the discovery sample to enable effect size comparison and to model with affective and anxiety symptoms as covariates. Outliers in neural activity were identified using a Z-score method (±3 SD) and excluded. Extracted beta values were then harmonized using neuroCombat in R (version 4.4) to adjust for scanner-related variance. Linear regression models were first performed using extracted beta values as independent variables and impulsivity facets as dependent variables in each sample, controlling for age, sex, and IQ. These linear models were repeated with and without affective and anxiety symptom covariates. Depressive symptoms (HRSD) were specified a priori as the primary covariate based on prior evidence linking depression to altered expression of impulsivity facets, whereas other symptoms (HAMA and YMRS) were examined in secondary analyses.

Following replication analyses, the discovery and replication samples were merged to estimate effect sizes of significant associations that replicated across both independent samples in a larger combined sample. Linear regression models identical to those used within individual samples were conducted in the combined dataset, again using extracted beta values as independent variables and impulsivity facets as dependent variables, controlling for age, sex, and IQ. These models were repeated with and without affective and anxiety symptom covariates (a priori HRSD, followed by HAMA and YMRS) to assess the robustness and specificity of associations across the combined cohort. To examine whether affective or anxiety symptoms were directly associated with neural correlates of impulsivity facets, zero-order correlations were calculated between extracted beta values of neural activity and affective and anxiety symptom severity scores in the combined sample. As a second internal model robustness check, k-fold cross-validation (5-fold) was conducted within the discovery sample using the same regression model specifications (i.e., beta values of neural activity predicting impulsivity facets with age, sex, IQ, and affective or anxiety symptom severity as covariates) to obtain out-of-sample estimates of model stability relevant to the combined sample.

### Aim 3: Test interaction effects

Interaction models were then estimated to test whether affective or anxiety symptoms moderated the associations between neural correlates and impulsivity facets within the combined sample. Linear regression models included the extracted beta values of neural activity associated with impulsivity facets identified in Aims 1-2, the relevant affective or anxiety symptom scale (i.e., HRSD, HAMA, YMRS), their interaction, as well as age, sex, and IQ as covariates. To increase statistical power, the interaction models were tested only in the combined dataset, with cohort entered as a covariate to account for any sample differences other than demographic and clinical factors. Model fit was evaluated by comparing additive and interaction models in the combined dataset using nested ANOVA tests to determine whether inclusion of the interaction term significantly improved model fit.

### Aim 4: Group comparisons

Participants meeting lifetime criteria for any externalizing disorder, as defined by SCID-5-RV modules, were grouped into a single externalizing disorders category from the combined sample. Externalizing disorders in this sample included ADHD (*n* = 19), oppositional defiant disorder (ODD; *n* = 18), conduct disorder (*n* = 1), intermittent explosive disorder (IED; *n* = 3), as well as a previous history of SUDs involving alcohol (*n* = 20), cannabis (*n* = 13), cocaine (*n* = 1), and other hallucinogens (*n* = 1). No participants met lifetime criteria for antisocial personality, gambling disorder, or history of other SUDs. In total, 58 participants met criteria for one or more externalizing disorders across the combined sample.

The remaining participants without externalizing disorders were stratified into tertiles based on impulsivity facet scores shown to have significant associations with neural activity during RE in the aforementioned analyses, forming low-, medium-, and high-impulsivity groups. Group differences in neural activity were examined using analysis of covariance (ANCOVA), controlling for age, sex, and IQ, followed by Tukey’s Honestly Significant Difference (HSD) post hoc tests to evaluate pairwise differences among the impulsivity tertile groups and the externalizing disorders group. A fifth group, comprising euthymic BD, was then added and ANCOVA models and Tukey’s HSD were re-estimated. Sensitivity analyses examined the effect of medication-use in the BD group on neural activity.

### Exploratory analyses

Exploratory whole-brain analyses were conducted in the replication sample to examine associations among neural activity and impulsivity facets identified in the discovery sample using identical preprocessing and thresholding procedures as in Aim 1. For any unique clusters identified in the replication sample, follow-up models with extracted beta values were used, as in Aim 2, to test associations among neural activity and impulsivity facets in each sample, and the impact of these association with affective or anxiety symptoms as covariates, as in Aim 3, to test potential moderating effects of these symptoms on neural activity-impulsivity facet associations.

## Results

### Aim 1 results: Identifying neural correlates of impulsivity facets in the discovery sample

**Fig. 1:**
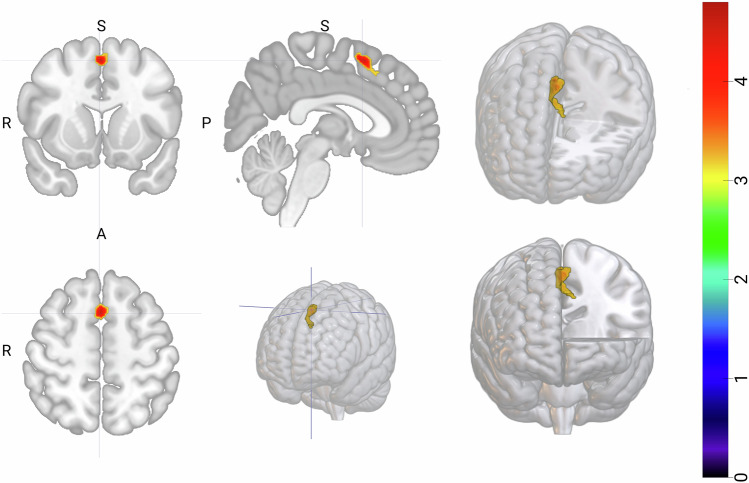
Thresholded cluster in the pre-supplementary motor area (pre-SMA) identified via whole-brain regression, showing a positive association with Fun Seeking during reward expectancy (RE). The color bar represents voxel-wise *t*-values. This cluster, visualized using MRIcroGL, was used as a mask for beta value extraction in subsequent analyses.

Whole-brain regressions revealed significant RE-related neural activity for Fun Seeking and Sensation Seeking in the positive contrasts. No significant clusters were found for the other impulsivity facets or in the negative contrasts. Fun Seeking was positively associated with activity in the pre-supplementary motor area (pre-SMA; *p*_FWE_ = 0.003, *k* = 167; Fig. 1), while Sensation Seeking was positively associated with activity in the right ventrolateral prefrontal cortex (R-vlPFC; *p*_FWE_ = 0.001, *k* = 194) and right superior frontal gyrus (SFG; *p*_FWE_ = 0.006, *k* = 151; full results in Table 2). Sensitivity analyses excluding IQ reduced the significance of the right SFG cluster below the Bonferroni-corrected threshold (*p*_FWE_ = 0.019). In contrast, the pre-SMA (*p*_FWE_ = 0.002) and R-vlPFC (*p*_FWE_ = 0.004) clusters remained significant.

**Table 2:** Whole-brain regression cluster activations during reward expectancy (RE) positively associated with Fun Seeking and Sensation Seeking.

Region	Laterality	MNI coordinates			No. of voxels in cluster	*t* Value	Cluster-level family-wise error (FWE) corrected *p* value
		x	y	z			
**BAS Fun Seeking (N** = **143)**							
Pre-supplementary motor area (BA 6)	Medial	0	14	54	167	4.75	0.003
	Left	−4	24	42		3.63	
	Left	−8	32	40		3.61	
Inferior frontal gyrus (BA 45, 47)	Right	40	22	14	78	4.60	0.112
	Right	38	10	8		3.51	
Dorsal anterior cingulate cortex (BA 32)	Right	10	2	40	29	4.36	0.802
Pre-supplementary motor area (BA 6)	Left	−16	6	68	63	4.36	0.206
Pre-supplementary motor area (BA 6)	Right	34	−4	42	77	4.15	0.117
Pre-supplementary motor area (BA 6)	Right	20	2	52	19	4.13	0.960
Putamen (BA 47)	Right	32	−2	10	36	4.04	0.650
	Right	28	−8	16		3.61	
Anterior prefrontal cortex (BA 10)	Left	−18	56	10	12	4.01	0.996
Inferior frontal gyrus (BA 45)	Left	−56	32	6	14	3.93	0.991
Primary motor cortex (BA 4)	Right	12	−22	68	12	3.90	0.996
Superior parietal lobule (BA 7)	Right	6	−70	52	23	3.86	0.911
Insula (BA 13)	Left	−36	2	2	18	3.86	0.969
Pre-supplementary motor area (BA 6)	Left	−10	0	58	6	3.78	1.000
Inferior frontal gyrus (BA 45)	Right	52	36	4	24	3.76	0.895
Anterior prefrontal cortex (BA 10)	Right	46	46	−8	42	3.75	0.522
Angular gyrus (BA 39)	Left	−60	−48	28	19	3.74	0.960
Pre-supplementary motor area (BA 6)	Right	18	6	64	23	3.73	0.911
Dorsal lateral prefrontal cortex (BA 9)	Right	10	50	38	14	3.69	0.991
*Remaining subthreshold clusters for Fun Seeking provided in Supplementary Materials							
**UPPS-P Sensation Seeking (N** = **143)**							
Ventral lateral prefrontal cortex (BA 47)	Right	50	42	−6	194	4.66	0.001
Superior frontal gyrus (BA 8)	Right	48	14	50	151	4.50	0.006
	Right	38	10	42		3.81	
	Right	34	2	38		3.75	
Pre-supplementary motor area (BA 6)	Right	18	6	62	33	4.37	0.717
Superior frontal gyrus (BA 8)	Right	14	38	50	17	3.88	0.977
Dorsal lateral prefrontal cortex (BA 9)	Medial	0	56	34	54	3.86	0.320
	Right	10	58	38		3.57	
	Left	−8	56	32		3.24	
Dorsal anterior cingulate cortex (BA 32)	Right	8	0	40	9	3.83	0.999
Angular gyrus (BA 39)	Right	46	−56	38	62	3.82	0.226
	Right	52	−56	32		3.70	
Anterior prefrontal cortex (BA 10)	Right	38	56	4	27	3.70	0.843
Angular gyrus (BA 39)	Left	−56	−56	46	18	3.64	0.969
Inferior frontal gyrus (BA 44)	Right	56	18	8	21	3.59	0.939
	Right	58	18	16		3.48	
Dorsal lateral prefrontal cortex (BA 46)	Left	−52	44	4	15	3.58	0.988
Middle temporal gyrus (BA 21)	Right	70	−26	−12	7	3.53	1.000
Fusiform gyrus (BA 37)	Right	42	−42	−14	5	3.52	1.000
Pre-supplementary motor area (BA 6)	Right	10	−22	68	4	3.44	1.000
Inferior frontal gyrus (BA 45)	Right	40	24	14	2	3.41	1.000

*BA*, Brodmann area; *MNI*, Montreal Neurological Institute.

### Aim 2 results: Replication via extracted beta values

**Fig. 2:**
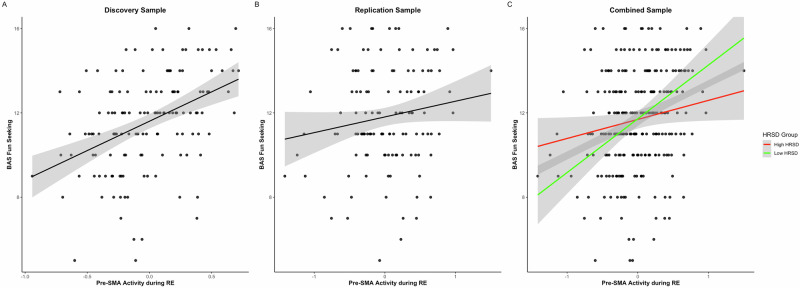
Pre-supplementary motor area activity during reward expectancy is positively associated with BAS Fun Seeking and moderated by depressive symptom severity. Associations between pre-supplementary motor area (pre-SMA) activity during reward expectancy (RE) and BAS Fun Seeking in the (**A**) discovery (*n* = 142), (**B**) replication (*N* = 122), and (**C**) combined (*N* = 264) samples. Each point represents an individual participant. Black lines depict linear regression fits with shaded 95% confidence intervals. For visual purposes only, in (**C**) the combined sample was divided into two groups based on a median split of depression symptom severity (Hamilton Rating Scale for Depression; HRSD), and a separate slope was fit for each group to visualize the pre-SMA × HRSD interaction: low HRSD = green and high HRSD = red.

Linear regressions were conducted using beta values extracted from significant clusters identified in the whole-brain regressions. All linear regression assumptions of normality, homoscedasticity, and multicollinearity were met. Greater pre-SMA activity significantly predicted higher Fun Seeking scores when depressive symptoms (HRSD) were included as a covariate in both the discovery (*β* = 2.73, SE = 0.51, 95% CI [1.72, 3.73], *p* < 0.001; Fig. 2A) and replication samples (*β* = 0.88, SE = 0.42, 95% CI [0.06, 1.71], *p* = 0.036; Fig. 2B). Without depressive symptom severity as a covariate, the association was significant in the discovery sample (*β* = 2.76, SE = 0.50, 95% CI [1.77, 3.76], *p* < 0.001) but not in the replication sample (*β* = 0.75, SE = 0.43, 95% CI [−0.10, 1.60], *p* = 0.082), indicating that higher levels of depressive symptoms likely attenuated the association between pre-SMA activity and Fun Seeking in the replication sample. Models substituting HAMA or YMRS for HRSD did not yield significant associations in the replication sample, indicating that depressive, but not manic/hypomanic or anxiety symptoms, attenuated the association between pre-SMA activity and Fun Seeking.

Although greater R-vlPFC activity significantly predicted higher Sensation Seeking in the discovery sample, both without and with depressive symptom severity as a covariate (*β* = 0.41, SE = 0.09, 95% CI [0.23, 0.59], *p* < 0.001; with HRSD: *β* = 0.41, SE = 0.09, 95% CI [0.23, 0.59], *p* < 0.001), the association did not replicate in the independent sample, regardless of the inclusion of depressive symptom severity as a covariate or not (*β* = 0.02, SE = 0.09, 95% CI [−0.16, 0.20], *p* = 0.833; with HRSD: *β* = 0.02, SE = 0.09, 95% CI [−0.15, 0.19], *p* = 0.849). Notably, in the replication sample, HRSD was inversely associated with Sensation Seeking (*β* = −0.03, SE = 0.009, 95% CI [−0.05, −0.01], *p* = 0.001), indicating that non-replication was not explained by unmodeled depressive symptoms. Models substituting HAMA or YMRS for HRSD did not yield significant associations in the replication sample.

Given replication of the association between greater pre-SMA activity and higher Fun Seeking scores across both independent samples when controlling for HRSD, these models were estimated in a combined sample. In the combined sample (*N* = 264), greater pre-SMA activity remained robustly associated with higher Fun Seeking when controlling for age, sex, IQ, and HRSD scores (*β* = 1.49, SE = 0.33, 95% CI [0.84, 2.13], *p* < 0.001), as well as in the model without HRSD (*β* = 1.44, SE = 0.33, 95% CI [0.80, 2.09], *p* < 0.001; Fig. 2C). Depressive symptom severity was not directly related to pre-SMA activity, as indicated by a nonsignificant zero-order correlation between beta values of pre-SMA activity and HRSD scores in the combined sample (*r* = 0.09, *p* = 0.149). Internal cross-validation indicated model stability (cross-validated partial *R*² = 0.16; Cohen’s *f*²=0.22) for the pre-SMA–Fun Seeking association including age, sex, IQ, and HRSD as covariates, indicating that the discovery sample as a whole did not solely drive the effect observed in the combined sample.

### Aim 3 results: Interaction effects

In the combined sample, a significant pre-SMA × HRSD interaction was observed (*β* = −0.08, SE = 0.040, 95% CI [−0.16, −0.01], *p* = 0.037), accompanied by a direct positive association between pre-SMA activity (*β* = 2.35, SE = 0.51, 95% CI [1.35, 3.36], *p* < 0.001) and a direct negative association between HRSD (*β* = −0.07, SE = 0.03, 95% CI [−0.12, −0.01], *p* = 0.019) and Fun Seeking. Model comparison via ANOVA confirmed that inclusion of the pre-SMA × HRSD interaction significantly improved model fit relative to the additive model (F(1,256) = 4.41, *p* = 0.037).

### Aim 4 results: group comparisons

Four groups were compared: low (*n* = 69), medium (*n* = 69), and high (*n* = 68) tertiles of Fun Seeking scores among participants without externalizing disorders, and a fourth group comprising participants with a lifetime externalizing disorder diagnosis (*n* = 58). ANCOVA revealed a significant main effect of group on pre-SMA activity (F(3, 257) = 7.06, *p* < 0.001). Residuals were normally distributed (Shapiro–Wilk *p* = 0.375), and assumptions of homogeneity of regression slopes were satisfied. Tukey’s HSD post hoc tests indicated significantly greater pre-SMA activity in the high-Fun Seeking group relative to the low-Fun Seeking group (*p* < 0.001) and in the externalizing disorders group relative to the low-Fun Seeking group (*p* = 0.017). No other group differences reached statistical significance.

When a fifth group comprising euthymic individuals with BD was added, ANCOVA again revealed a significant main effect of group on pre-SMA activity (F(4, 292) = 6.07, *p* < 0.001). Residuals were normally distributed (Shapiro–Wilk *p* = 0.495), and assumptions of homogeneity of regression slopes were satisfied. Tukey’s HSD comparisons showed greater pre-SMA activity in the high-Fun Seeking group compared with both the low-Fun Seeking group (*p* < 0.001) and the BD group (*p* = 0.018), as well as greater activity in the externalizing disorders group relative to the low-Fun Seeking group (*p* = 0.039). No other pairwise differences were significant.

Supplementary analyses, in which drug classes (i.e., antidepressant, benzodiazepine, mood stabilizers, or antipsychotics) were entered as binary covariates, suggested that lower pre-SMA activity in the BD group relative to the high-Fun Seeking group might have been the result of medication use in the BD group, particularly benzodiazepines, given a negative relationship between benzodiazepine use and pre-SMA activity (*β* = –0.87, *p* = 0.012), with a trend-level negative relationship observed with mood stabilizers (*β* = –0.29, *p* = 0.083). Full results are available in the Supplementary Materials.

To evaluate potential medication effects, the five-group ANCOVA model was re-estimated after excluding BD participants taking benzodiazepines. The main effect of group remained significant (F(4, 290) = 5.68, *p* < 0.001). Tukey’s HSD indicated that pre-SMA activity was significantly greater in the high-Fun Seeking group compared with the low-Fun Seeking group (*p* < 0.001) and in the externalizing disorders group compared with the low-Fun Seeking group (*p* = 0.035). The difference between the high-Fun Seeking and BD groups was attenuated and only marginally significant (*p* = 0.048). No other pairwise differences reached significance.

**Fig. 3:**
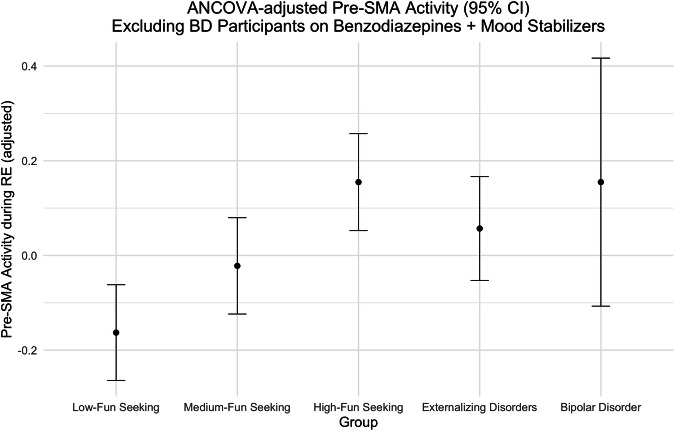
Estimated marginal means of pre–supplementary motor area (pre–SMA) activity during reward expectancy (RE) across five groups, adjusted for age, sex, and IQ using ANCOVA. Groups reflect tertiles of BAS Fun Seeking (low, medium, high), participants with externalizing disorders, and euthymic individuals with bipolar disorder (BD; medication-adjusted sample, excluding individuals on benzodiazepines and mood stabilizers). Error bars indicate 95% confidence intervals.

To further examine the influence of psychotropic medication, the model was re-estimated after excluding BD participants taking either benzodiazepines or mood stabilizers. The main effect of group remained significant (F(4, 266) = 5.64, *p* < 0.001). The high-Fun Seeking group continued to show greater pre-SMA activity than the low-Fun Seeking group (*p* < 0.001), as did the externalizing-disorders group compared to the low-Fun Seeking group (*p* = 0.028). The BD group no longer differed significantly from any other group (*p* ≥ 0.160), with estimated marginal means trending upward and comparable to the high-Fun Seeking group (BD = 0.155, high-Fun Seeking = 0.155; Fig. 3).

### Exploratory analyses

Given the significant relationship between pre-SMA activity and Fun Seeking across Aims 1–3, an exploratory whole-brain regression of Fun Seeking was conducted in the replication sample (*N* = 122). Activity during RE was observed in the pre-SMA, although it did not reach cluster-level significance. Full results are available in the Supplementary Materials.

## Discussion

Elevated pre-SMA activity during RE emerged as a robust, replicable neural correlate of approach-related impulsivity, specifically via the BAS Fun Seeking facet. This relationship was moderated by depressive symptom severity, such that higher depression severity attenuated the association between pre-SMA activity and Fun Seeking, with depression severity being inversely associated with Fun Seeking, but not significantly associated with pre-SMA activity. These findings indicate that elevated pre-SMA activity during RE represents a trait-linked neural marker of approach-related impulsivity, and that higher levels of depression severity may mask the behavioral expression, but not the underlying neural vulnerability to Fun Seeking. Group comparisons further showed that pre-SMA activity was elevated in individuals with high-Fun Seeking and those with externalizing disorders relative to individuals with low-Fun Seeking; comparable elevations in pre-SMA activity were observed among euthymic BD participants after accounting for medication effects. Together, these findings identify elevated pre-SMA activity during RE as a candidate neural marker of approach-related impulsivity linked to vulnerability for both externalizing psychopathology and BD.

The pre-SMA facilitates internally guided decision-making, action switching, and inhibition of prepotent responses [62]. Elevated pre-SMA activity has been linked to impulsive risk-taking behaviors in a broad range of externalizing disorders, including gambling and addiction-related disorders [63, 64]. Emerging evidence also implicates the pre-SMA in BD: aberrant pre-SMA structure, function, and connectivity have been associated with elevated mania/hypomania symptoms and BD [65–67]. These previous reports support the present findings that elevated pre-SMA activity is associated with vulnerability to externalizing disorders in general, and also to BD, via associations with Fun Seeking.

The present findings also thereby extend prior work establishing L-vlPFC activity during RE as a robust neural marker of mania/hypomania and thus BD risk [33] by identifying elevated pre-SMA activity during RE as a second, reproducible neural target associated with BD vulnerability. Together, these findings suggest that at least two partially distinct yet robust neural markers, L-vlPFC and pre-SMA activity during RE, may index separate but convergent pathways conferring vulnerability to BD, potentially reflecting differential contributions of heightened reward sensitivity and approach-related impulsivity, respectively. Identification of these two neural markers strengthens the potential for developing multivariate, circuit-based neural markers of BD risk and early identification, as well as neural targets for intervention.

Repetitive transcranial magnetic stimulation of the pre-SMA has been shown to reduce risk-taking in individuals with high trait impulsivity, but to increase risk-taking in those with low trait impulsivity [64], suggesting that pre-SMA excitability may follow a U-shaped model in which both hyper- and hypoactivity drive maladaptive behaviors. Additionally, targeted transcranial magnetic stimulation-induced hypoactivation of the pre-SMA during the decision-making phase of a delay discounting task reduced preferences for larger, delayed rewards, further supporting the potential U-shaped model and implicating the pre-SMA in reward-based impulsive decision-making [68]. The present findings suggest that, within the potential U-shaped excitability framework, pre-SMA hyperactivation is the more relevant pattern for impulsivity-related externalizing disorders and BD risk. Although pre-SMA activity may, in principle, reflect either impulsive drive or compensatory control in RE context, the observed attenuation of the pre-SMA–Fun Seeking association with increasing depressive symptoms is more consistent with pre-SMA hyperactivation indexing approach-related impulsive drive (i.e., energization) whose behavioral expression is dampened by depressive state, rather than successful compensatory inhibition.

Although the pre-SMA has not been directly targeted in BD interventions, it is likely modulated indirectly through stimulation of connected prefrontal or sensorimotor regions. For example, continuous (i.e., inhibitory) theta burst stimulation of the somatosensory cortex reduced smaller, immediate reward choices on a delay discounting task in individuals with BD [69], an effect not observed following L-vlPFC or right dorsolateral prefrontal cortex (R-dlPFC) stimulation. Continuous theta burst stimulation directly targeting the pre-SMA reduced symptom severity and trait impulsivity in other externalizing disorders, including gambling and addiction-related disorders [70]. Continuous theta burst stimulation to the somatosensory cortex also increased beta-band coherence between the L-vlPFC and R-dlPFC, suggesting network-level modulation likely involving the pre-SMA [69, 71]. Supporting this network-level modulation, sequential bilateral repetitive transcranial magnetic stimulation targeting the dlPFC (right 1 Hz, inhibitory; left 10 Hz, excitatory) modulated pre-SMA activity, potentially underlying improvements in executive function and memory in BD [72]. Together with the present findings, this evidence suggests that the pre-SMA is a promising neuromodulatory target for individuals with increased risk for externalizing disorders and BD, as a result of having elevated levels of Fun Seeking.

Interestingly, benzodiazepine use in BD participants was associated with reduced pre-SMA activity. As benzodiazepines enhance GABAergic transmission [73], which suppresses pre-SMA hyperactivation [74], this finding supports evidence that reduced GABA function may contribute to elevated dopaminergic activity associated with externalizing disorders and BD [73–78]. Notably, some mood stabilizers also appeared to contribute to reduced pre-SMA activity, which is consistent with the fact that several mood stabilizers—including gabapentin, topiramate, and oxcarbazepine, which were present in the BD group—exert GABAergic or GABA-modulating effects [79].

Although the discovery, replication, and combined samples were well-powered, further replication is needed to assess generalizability. The cross-sectional design limits conclusions about temporal directionality, and longitudinal or interventional studies can determine the extent to which pre-SMA activity during RE prospectively indexes BD or externalizing risk. Although externalizing disorders were modeled as a transdiagnostic liability dimension, larger diagnosis-specific samples can help to disentangle shared versus disorder-specific pre-SMA engagement. Additional research can also examine the role of R-vlPFC activity during RE in impulsivity-related neural pathways relevant to BD, given its less robust predictive value.

## Conclusion

Greater pre-SMA activity during RE emerged as a robust and replicable neural correlate of BAS Fun Seeking, moderated by depression severity. This finding identifies pre-SMA hyperactivity as a trait-linked marker of approach-related impulsivity associated with vulnerability to BD and externalizing disorders. Together with prior evidence implicating L-vlPFC activity during RE in BD risk, these results highlight distinct yet convergent neural pathways that can serve as markers for early identification and targets for intervention.

## Supplementary information

Supplementary Materials

## Data Availability

De-identified data from this study are stored in a public repository in accordance with data sharing policies. Access may be granted upon reasonable request to the corresponding author.
