# Supplementary material for: Elevated pre-supplementary motor area activity during reward expectancy: An impulsivity-related neural marker of vulnerability to bipolar and externalizing disorders
Source: Mol Psychiatry. 2026 Mar 19;31(7):4113–23. doi: 10.1038/s41380-026-03528-0 (PMC13071937; doi:10.1038/s41380-026-03528-0)
Supplement: Supplementary file 1 — Supplementary Materials [file 41380_2026_3528_MOESM1_ESM.docx]

**Supplementary Materials**

**Elevated Pre-Supplementary Motor Area Activity during Reward Expectancy: An Impulsivity-related Neural Marker of Vulnerability to Bipolar and Externalizing Disorders**

Raeder et al.

Table of Contents

[Methods 3](#_Toc218442330)

[Inclusion and Exclusion Criteria 3](#_Toc218442331)

[Key Measurements 4](#_Toc218442332)

[Additional Assessment Instruments 5](#_Toc218442333)

[Neuroimaging Acquisition Parameters 6](#_Toc218442334)

[Neuroimaging Data Acquisition 6](#_Toc218442335)

[Functional Imaging Task 6](#_Toc218442336)

[Figure 1. An outline of the phases in the reward task. 6](#_Toc218442337)

[Neuroimaging Data Preprocessing 7](#_Toc218442338)

[neuroCombat 7](#_Toc218442339)

[Intercorrelations Among BAS and UPPS-S Impulsivity Facets 8](#_Toc218442340)

[Whole-brain Regression Results of BAS Fun Seeking and UPPS-P Sensation Seeking in Discovery Sample 9](#_Toc218442341)

[Figure 2. BAS Fun Seeking Whole-brain Regression 19](#_Toc218442342)

[Figure 3. UPPS-P Sensation Seeking Whole-brain Regression 20](#_Toc218442343)

[Linear Regression Models of Pre-SMA Activity during RE and BAS Fun Seeking 21](#_Toc218442344)

[Linear Regression Models of R-vlPFC Activity during RE and UPPS-P Sensation Seeking 22](#_Toc218442345)

[Post Hoc Power Calculations 23](#_Toc218442346)

[Post Hoc Sensitivity Analysis 24](#_Toc218442347)

[Exploratory Analysis in BD Sample 25](#_Toc218442348)

[Medication Types as Predictors of Pre-SMA Activity in BD Sample 26](#_Toc218442349)

[Number of Manic/hypomanic Episodes as Predictors of Pre-SMA Activity in BD Sample 27](#_Toc218442350)

[Duration of Illness as Predictors of Pre-SMA Activity in BD Sample 28](#_Toc218442351)

[Effect of BD I versus II as Predictors of Pre-SMA Activity in BD Sample 29](#_Toc218442352)

[Exploratory Analysis: Whole-brain Regression in Replication Sample 30](#_Toc218442353)

[Figure 4. Significant clusters of neural activity associated with Fun Seeking in whole-brain regression in the replication sample. 30](#_Toc218442354)

[Linear Models for the Left Anterior Prefrontal Cortex and Fun Seeking 31](#_Toc218442355)

[Linear Models for the Precuneus and Fun Seeking 31](#_Toc218442356)

[Supplemental Discussion 31](#_Toc218442357)

[Figure 5. An overlay of the discovery sample whole-brain regression results (yellow) on top of the replication sample whole-brain regression results (green), showing overlapping activity in the precuneus. 32](#_Toc218442358)

[References 33](#_Toc218442359)

# Methods

## Inclusion and Exclusion Criteria

**Participants without bipolar disorder were included with:** (1) no current or lifetime history of BD but could have a first-degree family history of BD, MDD, attention-deficit/hyperactivity disorder (ADHD), or anxiety disorders; (2) no psychotropic medication usage in the past three months (except for brief use under two weeks); and (3) representation of a full range of emotional dysregulation and trait impulsivity, assessed via BAS and UPPS-P scales, ensuring variability in mania/hypomania risk.

**Participants with bipolar disorder (BD) were included with:** (1) a diagnosis of Bipolar I Disorder or Bipolar II Disorder, with equal representation of each subtype, and euthymic or in remission for at least two months, confirmed by the Structured Clinical Interview for DSM-5 Research Version (SCID-5-RV);^1^ (2) a Hamilton Rating Scale for Depression (HAMD)^2^ score ≤7 (or >7 without being in a depressive episode); (3) a Young Mania Rating Scale (YMRS)^3^ score <10 (or ≥10 without being in a manic/hypomanic episode); (4) a score <4 on the delusions, hallucinations, unusual thought content, and conceptual disorganization items of the Positive and Negative Syndrome Scale (PANSS),^4^ given that it is probable that psychosis impacts measures of impulsivity.^5^ BD participants were required to be unmedicated for more than two months or using atypical antipsychotics, mood stabilizers, sedatives, stimulants, and/or antidepressants (given common usage in individuals with BD) for more than two months.

**Participants were excluded with:** (1) history of head trauma; (2) comorbid neurological or pervasive developmental disorders (e.g., autism spectrum disorders); (3) systemic medical conditions confirmed via medical records and self-report, including cancers, progressive endocrine disorders, cardiac conditions, or other major illnesses (e.g., renal disease, multiple sclerosis, cerebral palsy), as well as chronic or acute conditions requiring ongoing medication (e.g., chronic pain, recent surgery); (4) use of medication for an excluded medical condition; (5) current psychosis or a lifetime history of primary psychotic disorder; (6) cognitive impairment, defined as a Mini-Mental State Examination (MMSE) score <24 and a National Adult Reading Test (NART) estimated Intelligence Quotient (IQ) <85;^6,7^ (7) substance-use disorder (SUD) or alcohol/substance abuse or dependence (including nicotine) in the past three months, based on a Structured Clinical Interview for DSM-5 Research Version (SCID-5-RV)^1^ assessment and available psychiatric records—non-SUD cannabis use was not an exclusion criterion due to its prevalence among young adults; and (8) factors contraindicating magnetic resonance imaging (MRI) scanning, including corrected visual acuity worse than 20/40 on a Snellen chart, presence or history of metallic foreign objects in the body, positive pregnancy test or self-reported pregnancy, and claustrophobia.

## Key Measurements

**The Behavioral Activation System** (BAS)^8^ scale is a validated self-report measure designed to assess individual differences in approach motivation and reward hypersensitivity.^9^ Based on Gray’s reinforcement sensitivity theory,^10^ it evaluates the tendency to pursue goals, seek out pleasurable experiences, and respond to potential rewards. The BAS scale consists of three subscales: Drive, which demonstrates persistent goal-directed behavior; Fun Seeking, which captures impulsive tendencies toward spontaneous or exciting experiences; and Reward Responsiveness, which gauges the intensity of positive emotions experienced in response to reward stimuli.

**The Urgency, Premeditation, Perseverance, and Sensation Seeking–Positive Urgency** (UPPS-P)^11^ scale is a validated self-report measure designed to assess distinct facets of impulsivity.^12^ It assesses five dimensions: Negative Urgency, which measures the tendency to act impulsively in response to negative emotion; Lack of Premeditation, which quantifies difficulty considering consequences before acting; Lack of Perseverance, which estimates an inability to sustain attention on a goal or task; Sensation Seeking, which evaluates a preference for intense, thrilling, or high-risk experiences; and Positive Urgency, which reflects impulsive tendencies triggered by heightened positive emotions.

**The Structured Clinical Interview for DSM-5 Research Version (SCID-5-RV)**^1^ is a semi-structured interview designed to diagnose major DSM-5 disorders. It is widely used in both clinical and research settings to ensure reliable and valid diagnosis of mental health disorders. The SCID-5-RV covers a broad range of psychiatric conditions, allowing interviewers to systematically assess and confirm diagnostic criteria based on participants’ responses. This tool is essential for researchers conducting studies that require precise diagnostic categorizations. Lifetime major depressive disorder (MDD) diagnosis was coded as follows: 1 = no history of MDD, 2 = subthreshold symptoms, and 3 = full lifetime MDD diagnosis; all three levels were included as an ordinal covariate in all models.

**The Hamilton Rating Scale for Depression (HRSD)**^2^ is a clinician-administered assessment used to measure the severity of depression in individuals. It consists of 17 to 21 items, depending on the version, that assess mood, physical symptoms, and cognitive impairment. Each item is scored on a scale of 0 to 4 or 0 to 2, with higher total scores indicating more severe depression. The HAM-D is frequently used in clinical trials and practice to evaluate treatment outcomes.

**The Hamilton Anxiety Rating Scale (HAMA)**^13^ is a clinician-administered assessment used to measure the severity of anxiety symptoms. It consists of 14 items covering psychic (e.g., anxious mood, tension, fears, insomnia, cognitive symptoms) and somatic domains (e.g., muscular, sensory, cardiovascular, respiratory, gastrointestinal, genitourinary, autonomic) plus behavior during interview. Each item is scored 0–4 (0 = not present to 4 = very severe), yielding a total score from 0–56, with higher scores indicating greater anxiety severity. The HAMA is widely used in clinical trials and routine practice to evaluate symptom burden and treatment response.

**The Young Mania Rating Scale (YMRS)**^3^ is a clinician-administered tool designed to assess the severity of manic symptoms in individuals with bipolar disorder. The scale consists of 11 items that evaluate mood, motor activity/energy, sexual interest, sleep, irritability, speech, language/thought disorder, content, disruptive/aggressive behavior, appearance, and insight. Each item is scored based on the severity and frequency of symptoms, with higher scores indicating more severe mania. The YMRS is widely used in both clinical settings and research to monitor treatment efficacy and symptom progression.

**The National Adult Reading Test (NART)**^7^ **IQ Estimate** is used to estimate premorbid intelligence levels in adults by assessing their ability to pronounce irregularly spelled words. The test comprises 50 words that are read aloud by the participant. Since the words do not follow typical phonetic rules, the ability to pronounce them correctly is believed to reflect prior educational attainment and intelligence. The NART is particularly useful in neuropsychological assessments where an estimate of an individual’s cognitive baseline is needed.

## Additional Assessment Instruments

**The Positive and Negative Syndrome Scale (PANSS)**^4^ is a clinician-administered instrument used to measure the severity of positive, negative, and general psychopathology symptoms in individuals with schizophrenia. The scale consists of 30 items, each rated on a 7-point scale, with higher scores indicating greater symptom severity. The PANSS is divided into three subscales: Positive Scale, Negative Scale, and General Psychopathology Scale. It is a standard tool in both clinical practice and research for assessing the treatment effects and symptomatology in schizophrenia.

**The Mini-Mental State Examination (MMSE)**^6^ is a widely used cognitive screening tool that assesses cognitive function across several domains: orientation, registration, attention and calculation, recall, language, and the ability to follow simple commands. The test is administered by asking a series of questions and performing simple tasks. The MMSE is scored out of 30 points, with higher scores indicating better cognitive function. Scores below 24 often suggest cognitive impairment, though this cutoff can vary depending on educational background and age.

## Neuroimaging Acquisition Parameters

### Neuroimaging Data Acquisition

Neuroimaging data were collected using three 3.0 Tesla Siemens Prisma MRI scanners at the University of Pittsburgh — one for the discovery sample and two for the replication sample. Functional blood oxygenation level-dependent (BOLD) T2*-weighted images were acquired using a multiband multi-echo echo-planar imaging (EPI) sequence. This sequence captured 70 oblique slices (12x multiband factor 5) with the following parameters: repetition time (TR) of 1500 ms, echo times (TE1=15.60 ms, TE2=39.78 ms, TE3=63.96 ms), slice thickness of 2 mm, field of view (FOV) of 210x210 mm, matrix size of 96x96, flip angle of 70°, and bandwidth of 2290 Hz/Px. Additionally, high-resolution structural images were collected using a 3D sagittal MPRAGE sequence (TR/TE=1520/3.17 ms; FOV=256x256 mm; flip angle=8°; voxel size of 1 mm³ isotropic; 176 continuous slices). Fieldmaps were also acquired with a TR of 731 ms, TE1 of 4.92 ms, TE2 of 7.38 ms, flip angle of 50°, and bandwidth of 566 Hz/Px.

### Functional Imaging Task

As previously outlined in other studies,^14^ the reward task is a 16-minute card-guessing game used to assess neural activity during the anticipation and receipt of a monetary reward. The task is structured into four distinct phases (Figure 1): (1) the choice phase, where the participant is prompted to guess whether a random card draw will be higher/lower than 5; (2) the anticipation (reward-expectancy; RE) phase, where an expectancy cue is presented (2-6 seconds, jittered) indicating whether the outcome may result in a monetary reward/loss; (3) the numerical feedback phase, where the result of the card draw is presented; and (4) the outcome, where the trial may yield a monetary win, loss, or remain neutral. Participants were given an opportunity to practice the task prior to the scan and were instructed that the outcome of each trial may result in actual monetary rewards ($1 per correct guess), losses (-75 cents per incorrect guess), or no monetary change. The task was divided into two segments, each lasting 8 minutes and consisting of 48 trials. There were 12 trials of each trial type and a 50% chance of each outcome, randomly presented with predetermined outcomes, thus ensuring statistical robustness and preventing expectancy biases.

### Figure 1. An outline of the phases in the reward task.

The anticipation phase triggers a reward expectancy (RE) cue, indicating whether the outcome will result in a monetary win/loss.

## Neuroimaging Data Preprocessing

The preprocessing of neuroimaging data was carried out using a combination of fMRIPrep, FSL (version 5.0.9), and AFNI (version 20160207). Initially, the first volume of the BOLD time series was extracted from the shortest echo and skull-stripped using fMRIPrep. This was followed by co-registration to the T1-weighted structural image using FreeSurfer’s bbregister tool. Head motion parameters were estimated with FSL's MCFLIRT. Slice-timing correction was performed using AFNI’s 3dTshift. The BOLD time series was then resampled to the native space. Multi-echo independent components analysis (ME-ICA) was applied using the Tedana tool to integrate multi-echo data. Additional preprocessing steps included despiking with AFNI’s 3dDespike and applying spatial smoothing with a 6 mm full width at half-maximum (FWHM) kernel.

For each participant, a first-level fixed-effect general linear model was implemented using Statistical Parametric Mapping, Version 12 (SPM12; Wellcome Centre for Human Neuroimaging). The primary regressor of interest, RE, was modeled as a parametric modulator during the anticipation phase (lasting 2–6 seconds). This regressor represented the expected value of the arrow in different conditions. The RE value was set to +0.5 for the potential win condition (50% chance of winning $1), –0.375 for the potential loss condition (50% chance of losing $0.75), +0.125 for the mixed condition (50% chance of winning $1 and 50% chance of losing $0.75), and zero for the neutral condition.

Motion-related quality control was assessed using frame-wise displacement (FWD) values obtained from fMRIPrep confound outputs. Participants were excluded if they exhibited a maximum FWD greater than 5 mm or a mean FWD greater than 0.5 mm, consistent with prior recommendations to minimize motion-related artifacts in BOLD signal.

## neuroCombat

To control for scanner-related variability in the replication sample due to the use of two MRI scanners, beta values were harmonized using the neuroCombat^15^ algorithm implemented in R. This empirical Bayes method adjusts for non-biological variance across batches while preserving signal associated with biological variables of interest. Scanner was specified as the batch variable, and age, sex, and IQ were included as covariates in the harmonization model. Harmonization was conducted independently for all extracted values in the replication sample prior to statistical modeling.

# Intercorrelations Among BAS and UPPS-S Impulsivity Facets

|  | **BAS Fun Seeking** | **BAS Drive** | **BAS Reward Responsiveness** | **Negative Urgency** | **Lack of Premeditation** | **Lack of Perseverance** | **Sensation Seeking** | **Positive Urgency** |
| --- | --- | --- | --- | --- | --- | --- | --- | --- |
| **BAS Fun Seeking** | 1.00 | 0.50 | 0.43 | 0.39 | 0.33 | 0.10 | 0.65 | 0.35 |
| **BAS Drive** | 0.50 | 1.00 | 0.50 | 0.15 | 0.03 | −0.19 | 0.43 | 0.20 |
| **BAS Reward Responsiveness** | 0.43 | 0.50 | 1.00 | 0.03 | −0.16 | −0.15 | 0.18 | −0.04 |
| **Negative Urgency** | 0.39 | 0.15 | 0.03 | 1.00 | 0.40 | 0.49 | 0.30 | 0.70 |
| **Lack of Premeditation** | 0.33 | 0.03 | −0.16 | 0.40 | 1.00 | 0.60 | 0.36 | 0.39 |
| **Lack of Perseverance** | 0.10 | −0.19 | −0.15 | 0.49 | 0.60 | 1.00 | 0.02 | 0.31 |
| **Sensation Seeking** | 0.65 | 0.43 | 0.18 | 0.30 | 0.36 | 0.02 | 1.00 | 0.39 |
| **Positive Urgency** | 0.35 | 0.20 | −0.04 | 0.70 | 0.39 | 0.31 | 0.39 | 1.00 |

# Whole-brain Regression Results of BAS Fun Seeking and UPPS-P Sensation Seeking in Discovery Sample

| **BAS - Fun Seeking - Positive Contrast** | | | | | | | | | | | | | |
| --- | --- | --- | --- | --- | --- | --- | --- | --- | --- | --- | --- | --- | --- |
| **Set-Level** | | **Cluster-Level** | | | | **Peak-Level** | | | | | **mm Coordinates** | | |
| *p* | *c* | p(FWE-corr) | p(FDR-corr) | equivk | p(unc) | p(FWE-corr) | p(FDR-corr) | *T* | equivZ | p(unc) | *x* | *y* | *z* |
| **0.000** | 68 | **0.003** | **0.008** | **167** | **0.000** | **0.197** | **0.567** | **4.75** | **4.57** | **0.000** | **0** | **14** | **54** |
|  |  |  |  |  |  | **1.000** | **0.879** | **3.63** | **3.54** | **0.000** | **-4** | **24** | **42** |
|  |  |  |  |  |  | **1.000** | **0.879** | **3.61** | **3.52** | **0.000** | **-8** | **32** | **40** |
|  |  | 0.112 | 0.105 | 78 | 0.004 | 0.315 | 0.567 | 4.60 | 4.43 | 0.000 | 40 | 22 | 14 |
|  |  |  |  |  |  | 1.000 | 0.935 | 3.51 | 3.43 | 0.000 | 38 | 10 | 8 |
|  |  | 0.802 | 0.590 | 29 | 0.061 | 0.574 | 0.660 | 4.36 | 4.22 | 0.000 | 10 | 2 | 40 |
|  |  | 0.206 | 0.147 | 64 | 0.009 | 0.586 | 0.660 | 4.36 | 4.21 | 0.000 | -16 | 6 | 68 |
|  |  | 0.117 | 0.105 | 77 | 0.005 | 0.820 | 0.879 | 4.15 | 4.02 | 0.000 | 34 | -4 | 42 |
|  |  | 0.960 | 0.632 | 19 | 0.121 | 0.845 | 0.879 | 4.13 | 4.00 | 0.000 | 20 | 2 | 52 |
|  |  | 0.650 | 0.446 | 36 | 0.039 | 0.914 | 0.879 | 4.04 | 3.92 | 0.000 | 32 | -2 | 10 |
|  |  |  |  |  |  | 1.000 | 0.879 | 3.61 | 3.52 | 0.000 | 28 | -8 | 16 |
|  |  | 0.996 | 0.717 | 12 | 0.211 | 0.933 | 0.879 | 4.01 | 3.89 | 0.000 | -18 | 56 | 10 |
|  |  | 0.991 | 0.713 | 14 | 0.178 | 0.966 | 0.879 | 3.93 | 3.82 | 0.000 | -56 | 32 | 6 |
|  |  | 0.996 | 0.717 | 12 | 0.211 | 0.975 | 0.879 | 3.90 | 3.79 | 0.000 | 12 | -22 | 68 |
|  |  | 0.911 | 0.616 | 23 | 0.091 | 0.984 | 0.879 | 3.86 | 3.76 | 0.000 | 6 | -70 | 52 |
|  |  | 0.969 | 0.632 | 18 | 0.130 | 0.985 | 0.879 | 3.86 | 3.75 | 0.000 | -36 | 2 | 2 |
|  |  | 1.000 | 0.743 | 6 | 0.375 | 0.995 | 0.879 | 3.78 | 3.68 | 0.000 | -10 | 0 | 58 |
|  |  | 0.895 | 0.616 | 24 | 0.085 | 0.996 | 0.879 | 3.76 | 3.66 | 0.000 | 52 | 36 | 4 |
|  |  | 0.522 | 0.377 | 42 | 0.028 | 0.997 | 0.879 | 3.75 | 3.65 | 0.000 | 46 | 46 | -8 |
|  |  | 0.960 | 0.632 | 19 | 0.121 | 0.997 | 0.879 | 3.74 | 3.65 | 0.000 | -60 | -48 | 28 |
|  |  | 0.911 | 0.616 | 23 | 0.091 | 0.998 | 0.879 | 3.73 | 3.64 | 0.000 | 18 | 6 | 64 |
|  |  | 0.991 | 0.713 | 14 | 0.178 | 0.999 | 0.879 | 3.69 | 3.60 | 0.000 | 10 | 50 | 38 |
|  |  | 1.000 | 0.743 | 7 | 0.338 | 1.000 | 0.879 | 3.64 | 3.55 | 0.000 | -42 | -16 | 28 |
|  |  | 0.969 | 0.632 | 18 | 0.130 | 1.000 | 0.879 | 3.64 | 3.55 | 0.000 | 48 | 16 | 0 |
|  |  | 1.000 | 0.743 | 6 | 0.375 | 1.000 | 0.879 | 3.63 | 3.54 | 0.000 | -32 | 8 | 8 |
|  |  | 1.000 | 0.743 | 7 | 0.338 | 1.000 | 0.879 | 3.63 | 3.54 | 0.000 | 34 | 2 | -36 |
|  |  | 1.000 | 0.743 | 4 | 0.473 | 1.000 | 0.879 | 3.61 | 3.52 | 0.000 | 42 | -44 | -14 |
|  |  | 0.996 | 0.717 | 12 | 0.211 | 1.000 | 0.879 | 3.60 | 3.52 | 0.000 | -12 | -52 | 64 |
|  |  | 1.000 | 0.743 | 8 | 0.305 | 1.000 | 0.879 | 3.57 | 3.49 | 0.000 | -20 | -62 | 12 |
|  |  | 1.000 | 0.743 | 6 | 0.375 | 1.000 | 0.879 | 3.57 | 3.48 | 0.000 | 36 | -4 | 18 |
|  |  | 0.999 | 0.743 | 9 | 0.277 | 1.000 | 0.879 | 3.56 | 3.48 | 0.000 | 6 | -52 | 62 |
|  |  | 1.000 | 0.743 | 3 | 0.539 | 1.000 | 0.879 | 3.56 | 3.48 | 0.000 | -30 | 4 | 14 |
|  |  | 0.983 | 0.688 | 16 | 0.152 | 1.000 | 0.935 | 3.51 | 3.43 | 0.000 | 44 | 0 | 48 |
|  |  | 1.000 | 0.743 | 4 | 0.473 | 1.000 | 0.935 | 3.48 | 3.40 | 0.000 | -8 | -48 | 54 |
|  |  | 1.000 | 0.743 | 4 | 0.473 | 1.000 | 0.935 | 3.47 | 3.39 | 0.000 | 6 | -16 | 78 |
|  |  | 1.000 | 0.743 | 3 | 0.539 | 1.000 | 0.935 | 3.47 | 3.39 | 0.000 | 18 | -58 | 18 |
|  |  | 1.000 | 0.743 | 3 | 0.539 | 1.000 | 0.935 | 3.43 | 3.36 | 0.000 | -12 | -22 | 36 |
|  |  | 1.000 | 0.743 | 1 | 0.743 | 1.000 | 0.935 | 3.43 | 3.35 | 0.000 | 50 | 0 | -28 |
|  |  | 1.000 | 0.743 | 2 | 0.624 | 1.000 | 0.935 | 3.42 | 3.35 | 0.000 | -6 | -24 | 50 |
|  |  | 1.000 | 0.743 | 3 | 0.539 | 1.000 | 0.935 | 3.42 | 3.34 | 0.000 | 20 | 48 | 26 |
|  |  | 1.000 | 0.743 | 7 | 0.338 | 1.000 | 0.935 | 3.39 | 3.32 | 0.000 | 14 | 58 | 26 |
|  |  | 1.000 | 0.743 | 5 | 0.420 | 1.000 | 0.935 | 3.39 | 3.31 | 0.000 | 48 | 12 | 50 |
|  |  | 1.000 | 0.743 | 1 | 0.743 | 1.000 | 0.935 | 3.38 | 3.31 | 0.000 | -32 | 42 | 18 |
|  |  | 1.000 | 0.743 | 4 | 0.473 | 1.000 | 0.948 | 3.37 | 3.30 | 0.000 | -22 | 14 | 2 |
|  |  | 1.000 | 0.743 | 7 | 0.338 | 1.000 | 0.951 | 3.36 | 3.29 | 0.001 | -36 | 54 | -14 |
|  |  | 1.000 | 0.743 | 4 | 0.473 | 1.000 | 0.951 | 3.35 | 3.28 | 0.001 | 12 | 68 | 6 |
|  |  | 1.000 | 0.743 | 3 | 0.539 | 1.000 | 0.955 | 3.33 | 3.26 | 0.001 | 0 | 62 | 34 |
|  |  | 1.000 | 0.743 | 2 | 0.624 | 1.000 | 0.955 | 3.32 | 3.25 | 0.001 | 18 | -26 | 54 |
|  |  | 1.000 | 0.743 | 7 | 0.338 | 1.000 | 0.955 | 3.31 | 3.24 | 0.001 | 8 | -64 | 60 |
|  |  | 1.000 | 0.743 | 5 | 0.420 | 1.000 | 0.955 | 3.30 | 3.24 | 0.001 | 12 | 16 | 64 |
|  |  | 1.000 | 0.743 | 3 | 0.539 | 1.000 | 0.955 | 3.30 | 3.23 | 0.001 | -18 | 50 | -12 |
|  |  | 1.000 | 0.743 | 3 | 0.539 | 1.000 | 0.955 | 3.29 | 3.22 | 0.001 | 18 | 0 | 42 |
|  |  | 1.000 | 0.743 | 4 | 0.473 | 1.000 | 0.955 | 3.29 | 3.22 | 0.001 | 58 | 20 | 6 |
|  |  | 1.000 | 0.743 | 2 | 0.624 | 1.000 | 0.955 | 3.28 | 3.21 | 0.001 | 0 | 28 | 54 |
|  |  | 1.000 | 0.743 | 6 | 0.375 | 1.000 | 0.961 | 3.26 | 3.19 | 0.001 | 2 | 50 | 26 |
|  |  | 1.000 | 0.743 | 1 | 0.743 | 1.000 | 0.961 | 3.25 | 3.19 | 0.001 | 4 | 12 | 40 |
|  |  | 1.000 | 0.743 | 2 | 0.624 | 1.000 | 0.961 | 3.24 | 3.18 | 0.001 | 4 | -52 | -2 |
|  |  | 1.000 | 0.743 | 2 | 0.624 | 1.000 | 0.961 | 3.24 | 3.17 | 0.001 | -22 | -6 | 42 |
|  |  | 1.000 | 0.743 | 1 | 0.743 | 1.000 | 0.961 | 3.22 | 3.15 | 0.001 | -24 | 64 | -2 |
|  |  | 1.000 | 0.743 | 1 | 0.743 | 1.000 | 0.961 | 3.22 | 3.15 | 0.001 | 34 | -52 | 50 |
|  |  | 1.000 | 0.743 | 1 | 0.743 | 1.000 | 0.961 | 3.22 | 3.15 | 0.001 | 30 | 30 | -18 |
|  |  | 1.000 | 0.743 | 2 | 0.624 | 1.000 | 0.961 | 3.21 | 3.15 | 0.001 | -58 | -48 | 36 |
|  |  | 1.000 | 0.743 | 1 | 0.743 | 1.000 | 0.961 | 3.20 | 3.14 | 0.001 | 20 | -6 | 42 |
|  |  | 1.000 | 0.743 | 1 | 0.743 | 1.000 | 0.961 | 3.19 | 3.13 | 0.001 | -10 | -18 | 76 |
|  |  | 1.000 | 0.743 | 1 | 0.743 | 1.000 | 0.961 | 3.19 | 3.13 | 0.001 | 28 | -60 | 30 |
|  |  | 1.000 | 0.743 | 1 | 0.743 | 1.000 | 0.961 | 3.18 | 3.12 | 0.001 | 44 | 20 | 24 |
|  |  | 1.000 | 0.743 | 1 | 0.743 | 1.000 | 0.961 | 3.18 | 3.12 | 0.001 | -26 | -6 | -2 |
|  |  | 1.000 | 0.743 | 1 | 0.743 | 1.000 | 0.961 | 3.18 | 3.12 | 0.001 | 10 | -12 | 72 |
|  |  | 1.000 | 0.743 | 1 | 0.743 | 1.000 | 0.961 | 3.18 | 3.12 | 0.001 | 8 | 62 | 24 |
|  |  | 1.000 | 0.743 | 1 | 0.743 | 1.000 | 0.961 | 3.18 | 3.12 | 0.001 | 52 | 26 | 2 |
|  |  | 1.000 | 0.743 | 2 | 0.624 | 1.000 | 0.962 | 3.17 | 3.11 | 0.001 | 70 | -26 | -12 |
|  |  | 1.000 | 0.743 | 1 | 0.743 | 1.000 | 0.981 | 3.16 | 3.10 | 0.001 | -14 | -58 | 30 |
| **BAS - Fun Seeking - Negative Contrast** | | | | | | | | | | | | | |
| **Set-Level** | | **Cluster-Level** | | | | **Peak-Level** | | | | | **mm Coordinates** | | |
| *p* | *c* | p(FWE-corr) | p(FDR-corr) | equivk | p(unc) | p(FWE-corr) | p(FDR-corr) | *T* | equivZ | p(unc) | *x* | *y* | *z* |
| **1.000** | 4 | 0.998 | 0.461 | 11 | 0.230 | 0.810 | 0.249 | 4.16 | 4.03 | 0.000 | 40 | -32 | 30 |
|  |  | 0.998 | 0.461 | 11 | 0.230 | 0.999 | 0.562 | 3.65 | 3.56 | 0.000 | 4 | -92 | -6 |
|  |  | 1.000 | 0.624 | 2 | 0.624 | 1.000 | 0.762 | 3.38 | 3.31 | 0.000 | 8 | 20 | -18 |
|  |  | 1.000 | 0.624 | 3 | 0.539 | 1.000 | 0.833 | 3.23 | 3.17 | 0.001 | 22 | -82 | -14 |
| **UPPS-P - Sensation Seeking - Positive Contrast** | | | | | | | | | | | | | |
| **Set-Level** | | **Cluster-Level** | | | | **Peak-Level** | | | | | **mm Coordinates** | | |
| *p* | *c* | p(FWE-corr) | p(FDR-corr) | equivk | p(unc) | p(FWE-corr) | p(FDR-corr) | *T* | equivZ | p(unc) | *x* | *y* | *z* |
| **0.022** | 38 | **0.001** | **0.002** | **194** | **0.000** | **0.267** | **0.488** | **4.66** | **4.48** | **0.000** | **50** | **42** | **-6** |
|  |  | **0.006** | **0.004** | **151** | **0.000** | **0.418** | **0.488** | **4.50** | **4.34** | **0.000** | **48** | **14** | **50** |
|  |  |  |  |  |  | **0.992** | **0.943** | **3.81** | **3.71** | **0.000** | **38** | **10** | **42** |
|  |  |  |  |  |  | **0.997** | **0.943** | **3.75** | **3.65** | **0.000** | **34** | **2** | **38** |
|  |  | 0.717 | 0.360 | 33 | 0.047 | 0.568 | 0.505 | 4.37 | 4.22 | 0.000 | 18 | 6 | 62 |
|  |  | 0.977 | 0.595 | 17 | 0.141 | 0.979 | 0.943 | 3.88 | 3.78 | 0.000 | 14 | 38 | 50 |
|  |  | 0.320 | 0.138 | 54 | 0.014 | 0.984 | 0.943 | 3.86 | 3.76 | 0.000 | 0 | 56 | 34 |
|  |  |  |  |  |  | 1.000 | 0.943 | 3.57 | 3.49 | 0.000 | 10 | 58 | 38 |
|  |  |  |  |  |  | 1.000 | 0.943 | 3.24 | 3.17 | 0.001 | -8 | 56 | 32 |
|  |  | 0.999 | 0.744 | 9 | 0.277 | 0.989 | 0.943 | 3.83 | 3.73 | 0.000 | 8 | 0 | 40 |
|  |  | 0.226 | 0.122 | 62 | 0.010 | 0.990 | 0.943 | 3.82 | 3.72 | 0.000 | 46 | -56 | 38 |
|  |  |  |  |  |  | 0.999 | 0.943 | 3.70 | 3.60 | 0.000 | 52 | -56 | 32 |
|  |  | 0.843 | 0.440 | 27 | 0.069 | 0.999 | 0.943 | 3.70 | 3.61 | 0.000 | 38 | 56 | 4 |
|  |  | 0.969 | 0.595 | 18 | 0.131 | 1.000 | 0.943 | 3.64 | 3.55 | 0.000 | -56 | -56 | 46 |
|  |  | 0.939 | 0.569 | 21 | 0.105 | 1.000 | 0.943 | 3.59 | 3.51 | 0.000 | 56 | 18 | 8 |
|  |  |  |  |  |  | 1.000 | 0.943 | 3.48 | 3.40 | 0.000 | 58 | 18 | 16 |
|  |  | 0.988 | 0.627 | 15 | 0.165 | 1.000 | 0.943 | 3.58 | 3.50 | 0.000 | -52 | 44 | 4 |
|  |  | 1.000 | 0.744 | 7 | 0.338 | 1.000 | 0.943 | 3.53 | 3.44 | 0.000 | 70 | -26 | -12 |
|  |  | 1.000 | 0.744 | 5 | 0.420 | 1.000 | 0.943 | 3.52 | 3.44 | 0.000 | 42 | -42 | -14 |
|  |  | 1.000 | 0.744 | 4 | 0.474 | 1.000 | 0.943 | 3.44 | 3.36 | 0.000 | 10 | -22 | 68 |
|  |  | 1.000 | 0.744 | 2 | 0.625 | 1.000 | 0.943 | 3.41 | 3.33 | 0.000 | 40 | 24 | 14 |
|  |  | 1.000 | 0.744 | 4 | 0.474 | 1.000 | 0.943 | 3.39 | 3.32 | 0.000 | 6 | -14 | 78 |
|  |  | 1.000 | 0.744 | 7 | 0.338 | 1.000 | 0.943 | 3.39 | 3.32 | 0.000 | 36 | 24 | 52 |
|  |  | 1.000 | 0.744 | 3 | 0.540 | 1.000 | 0.943 | 3.37 | 3.29 | 0.000 | 54 | -42 | 36 |
|  |  | 1.000 | 0.744 | 3 | 0.540 | 1.000 | 0.943 | 3.36 | 3.29 | 0.000 | 38 | 44 | -16 |
|  |  | 1.000 | 0.744 | 1 | 0.744 | 1.000 | 0.943 | 3.35 | 3.28 | 0.001 | -18 | -24 | 40 |
|  |  | 1.000 | 0.744 | 2 | 0.625 | 1.000 | 0.943 | 3.31 | 3.24 | 0.001 | 2 | -32 | -42 |
|  |  | 1.000 | 0.744 | 1 | 0.744 | 1.000 | 0.943 | 3.30 | 3.23 | 0.001 | -48 | -52 | 28 |
|  |  | 1.000 | 0.744 | 1 | 0.744 | 1.000 | 0.943 | 3.29 | 3.22 | 0.001 | -30 | 14 | 32 |
|  |  | 1.000 | 0.744 | 3 | 0.540 | 1.000 | 0.943 | 3.28 | 3.22 | 0.001 | 0 | -24 | 46 |
|  |  | 1.000 | 0.744 | 2 | 0.625 | 1.000 | 0.943 | 3.28 | 3.22 | 0.001 | 16 | 60 | 30 |
|  |  | 1.000 | 0.744 | 1 | 0.744 | 1.000 | 0.943 | 3.26 | 3.19 | 0.001 | -36 | 30 | -8 |
|  |  | 1.000 | 0.744 | 5 | 0.420 | 1.000 | 0.943 | 3.26 | 3.19 | 0.001 | 0 | 18 | 54 |
|  |  | 1.000 | 0.744 | 1 | 0.744 | 1.000 | 0.943 | 3.26 | 3.19 | 0.001 | 22 | 66 | -2 |
|  |  | 1.000 | 0.744 | 2 | 0.625 | 1.000 | 0.943 | 3.26 | 3.19 | 0.001 | -36 | -54 | -18 |
|  |  | 1.000 | 0.744 | 1 | 0.744 | 1.000 | 0.943 | 3.24 | 3.17 | 0.001 | -4 | 24 | 66 |
|  |  | 1.000 | 0.744 | 2 | 0.625 | 1.000 | 0.943 | 3.23 | 3.17 | 0.001 | 10 | -58 | 38 |
|  |  | 1.000 | 0.744 | 3 | 0.540 | 1.000 | 0.943 | 3.22 | 3.16 | 0.001 | -52 | 20 | 38 |
|  |  | 1.000 | 0.744 | 1 | 0.744 | 1.000 | 0.943 | 3.21 | 3.15 | 0.001 | -4 | 40 | 42 |
|  |  | 1.000 | 0.744 | 1 | 0.744 | 1.000 | 0.943 | 3.20 | 3.14 | 0.001 | -36 | 4 | 46 |
|  |  | 1.000 | 0.744 | 1 | 0.744 | 1.000 | 0.943 | 3.20 | 3.14 | 0.001 | 12 | 0 | 56 |
|  |  | 1.000 | 0.744 | 1 | 0.744 | 1.000 | 0.959 | 3.19 | 3.13 | 0.001 | 32 | -10 | 34 |
|  |  | 1.000 | 0.744 | 1 | 0.744 | 1.000 | 0.965 | 3.18 | 3.11 | 0.001 | -32 | 22 | 14 |
|  |  | 1.000 | 0.744 | 1 | 0.744 | 1.000 | 0.994 | 3.15 | 3.09 | 0.001 | -60 | -44 | 32 |
| **UPPS-P - Sensation Seeking - Negative Contrast** | | | | | | | | | | | | | |
| **Set-Level** | | **Cluster-Level** | | | | **Peak-Level** | | | | | **mm Coordinates** | | |
| *p* | *c* | p(FWE-corr) | p(FDR-corr) | equivk | p(unc) | p(FWE-corr) | p(FDR-corr) | *T* | equivZ | p(unc) | *x* | *y* | *z* |
| **0.999** | 13 | 0.586 | 0.430 | 39 | 0.033 | 0.573 | 0.415 | 4.37 | 4.22 | 0.000 | 6 | -92 | -8 |
|  |  | 1.000 | 0.738 | 5 | 0.420 | 0.998 | 0.776 | 3.72 | 3.62 | 0.000 | 8 | -34 | 8 |
|  |  | 1.000 | 0.738 | 3 | 0.540 | 1.000 | 0.776 | 3.63 | 3.54 | 0.000 | -24 | 6 | -28 |
|  |  | 1.000 | 0.738 | 5 | 0.420 | 1.000 | 0.776 | 3.56 | 3.48 | 0.000 | 28 | 8 | -30 |
|  |  | 0.999 | 0.738 | 9 | 0.277 | 1.000 | 0.776 | 3.52 | 3.44 | 0.000 | 36 | -4 | -26 |
|  |  | 1.000 | 0.738 | 5 | 0.420 | 1.000 | 0.776 | 3.48 | 3.40 | 0.000 | 10 | -6 | -10 |
|  |  | 1.000 | 0.738 | 2 | 0.625 | 1.000 | 0.776 | 3.40 | 3.33 | 0.000 | -8 | -2 | -12 |
|  |  | 1.000 | 0.738 | 4 | 0.474 | 1.000 | 0.776 | 3.40 | 3.33 | 0.000 | -10 | -86 | -18 |
|  |  | 1.000 | 0.738 | 4 | 0.474 | 1.000 | 0.776 | 3.33 | 3.26 | 0.001 | 34 | -58 | 0 |
|  |  | 1.000 | 0.744 | 1 | 0.744 | 1.000 | 0.776 | 3.33 | 3.26 | 0.001 | -36 | -16 | -8 |
|  |  | 1.000 | 0.738 | 2 | 0.625 | 1.000 | 0.776 | 3.30 | 3.23 | 0.001 | 12 | 14 | 52 |
|  |  | 1.000 | 0.738 | 2 | 0.625 | 1.000 | 0.776 | 3.29 | 3.23 | 0.001 | -12 | -28 | -14 |
|  |  | 1.000 | 0.744 | 1 | 0.744 | 1.000 | 0.935 | 3.18 | 3.12 | 0.001 | 10 | -34 | -36 |
| **BAS - Fun Seeking - Positive Contrast (NO IQ)** | | | | | | | | | | | | | |
| **Set-Level** | | **Cluster-Level** | | | | **Peak-Level** | | | | | **mm Coordinates** | | |
| *p* | *c* | p(FWE-corr) | p(FDR-corr) | equivk | p(unc) | p(FWE-corr) | p(FDR-corr) | *T* | equivZ | p(unc) | *x* | *y* | *z* |
| **0.000** | 68 | **0.002** | **0.006** | **175** | **0.000** | **0.136** | **0.347** | **4.86** | **4.66** | **0.000** | **0** | **14** | **54** |
|  |  |  |  |  |  | **0.997** | **0.794** | **3.74** | **3.64** | **0.000** | **4** | **18** | **48** |
|  |  |  |  |  |  | **1.000** | **0.841** | **3.58** | **3.50** | **0.000** | **0** | **26** | **48** |
|  |  | 0.056 | 0.074 | 94 | 0.002 | 0.207 | 0.347 | 4.74 | 4.55 | 0.000 | 40 | 22 | 12 |
|  |  |  |  |  |  | 0.999 | 0.819 | 3.69 | 3.60 | 0.000 | 38 | 10 | 10 |
|  |  | 0.269 | 0.199 | 58 | 0.012 | 0.678 | 0.634 | 4.28 | 4.14 | 0.000 | -14 | 4 | 66 |
|  |  | 0.925 | 0.632 | 22 | 0.097 | 0.742 | 0.634 | 4.22 | 4.09 | 0.000 | 20 | 2 | 52 |
|  |  | 0.861 | 0.629 | 26 | 0.074 | 0.743 | 0.634 | 4.22 | 4.09 | 0.000 | 32 | -2 | 10 |
|  |  | 0.781 | 0.590 | 30 | 0.057 | 0.772 | 0.634 | 4.20 | 4.06 | 0.000 | 10 | 2 | 40 |
|  |  |  |  |  |  | 1.000 | 0.956 | 3.43 | 3.36 | 0.000 | 18 | -2 | 44 |
|  |  | 0.216 | 0.199 | 63 | 0.009 | 0.955 | 0.794 | 3.96 | 3.85 | 0.000 | 34 | 0 | 40 |
|  |  | 0.998 | 0.743 | 11 | 0.230 | 0.964 | 0.794 | 3.94 | 3.83 | 0.000 | -18 | 56 | 10 |
|  |  | 0.950 | 0.632 | 20 | 0.112 | 0.969 | 0.794 | 3.92 | 3.81 | 0.000 | -36 | 0 | 2 |
|  |  | 0.802 | 0.590 | 29 | 0.061 | 0.974 | 0.794 | 3.91 | 3.80 | 0.000 | 16 | 6 | 64 |
|  |  | 0.991 | 0.714 | 14 | 0.178 | 0.985 | 0.794 | 3.86 | 3.75 | 0.000 | -56 | 32 | 6 |
|  |  | 1.000 | 0.743 | 8 | 0.305 | 0.991 | 0.794 | 3.82 | 3.72 | 0.000 | -10 | 0 | 58 |
|  |  | 0.896 | 0.632 | 24 | 0.085 | 0.995 | 0.794 | 3.78 | 3.68 | 0.000 | 48 | 16 | 0 |
|  |  | 0.999 | 0.743 | 9 | 0.277 | 0.995 | 0.794 | 3.78 | 3.68 | 0.000 | 36 | -4 | 18 |
|  |  | 0.960 | 0.632 | 19 | 0.121 | 0.996 | 0.794 | 3.76 | 3.67 | 0.000 | 6 | -70 | 50 |
|  |  | 0.969 | 0.633 | 18 | 0.130 | 0.996 | 0.794 | 3.76 | 3.66 | 0.000 | -60 | -48 | 28 |
|  |  | 0.999 | 0.743 | 10 | 0.252 | 0.997 | 0.794 | 3.74 | 3.65 | 0.000 | -42 | -16 | 28 |
|  |  | 0.996 | 0.743 | 12 | 0.211 | 0.997 | 0.794 | 3.73 | 3.64 | 0.000 | 10 | -22 | 68 |
|  |  | 0.950 | 0.632 | 20 | 0.112 | 0.998 | 0.794 | 3.73 | 3.64 | 0.000 | -32 | 8 | 8 |
|  |  | 0.991 | 0.714 | 14 | 0.178 | 0.998 | 0.794 | 3.73 | 3.63 | 0.000 | 10 | 48 | 38 |
|  |  | 1.000 | 0.743 | 7 | 0.338 | 0.999 | 0.819 | 3.69 | 3.60 | 0.000 | 34 | 2 | -36 |
|  |  | 0.672 | 0.568 | 35 | 0.042 | 1.000 | 0.823 | 3.64 | 3.55 | 0.000 | 46 | 46 | -8 |
|  |  | 0.983 | 0.689 | 16 | 0.152 | 1.000 | 0.823 | 3.63 | 3.54 | 0.000 | 52 | 36 | 4 |
|  |  | 0.999 | 0.743 | 10 | 0.252 | 1.000 | 0.823 | 3.63 | 3.54 | 0.000 | -20 | -62 | 12 |
|  |  | 1.000 | 0.743 | 4 | 0.473 | 1.000 | 0.823 | 3.62 | 3.53 | 0.000 | 42 | -44 | -14 |
|  |  | 1.000 | 0.743 | 7 | 0.338 | 1.000 | 0.841 | 3.59 | 3.50 | 0.000 | 18 | -58 | 18 |
|  |  | 0.999 | 0.743 | 9 | 0.277 | 1.000 | 0.841 | 3.58 | 3.49 | 0.000 | 6 | -52 | 62 |
|  |  | 1.000 | 0.743 | 5 | 0.420 | 1.000 | 0.884 | 3.55 | 3.46 | 0.000 | 28 | -8 | 16 |
|  |  | 0.999 | 0.743 | 9 | 0.277 | 1.000 | 0.921 | 3.50 | 3.42 | 0.000 | -12 | -50 | 64 |
|  |  | 1.000 | 0.743 | 4 | 0.473 | 1.000 | 0.956 | 3.39 | 3.32 | 0.000 | 44 | 0 | 48 |
|  |  | 1.000 | 0.743 | 1 | 0.743 | 1.000 | 0.956 | 3.38 | 3.31 | 0.000 | 50 | 0 | -28 |
|  |  | 1.000 | 0.743 | 5 | 0.420 | 1.000 | 0.956 | 3.38 | 3.31 | 0.000 | 58 | 20 | 6 |
|  |  | 1.000 | 0.743 | 3 | 0.539 | 1.000 | 0.956 | 3.36 | 3.29 | 0.001 | 20 | 48 | 26 |
|  |  | 1.000 | 0.743 | 1 | 0.743 | 1.000 | 0.956 | 3.34 | 3.27 | 0.001 | -18 | 50 | -12 |
|  |  | 1.000 | 0.743 | 6 | 0.375 | 1.000 | 0.956 | 3.33 | 3.26 | 0.001 | 12 | 16 | 64 |
|  |  | 1.000 | 0.743 | 1 | 0.743 | 1.000 | 0.956 | 3.33 | 3.26 | 0.001 | -32 | 42 | 18 |
|  |  | 1.000 | 0.743 | 3 | 0.539 | 1.000 | 0.956 | 3.33 | 3.26 | 0.001 | 0 | 28 | 54 |
|  |  | 1.000 | 0.743 | 4 | 0.473 | 1.000 | 0.956 | 3.32 | 3.26 | 0.001 | 24 | 58 | 8 |
|  |  | 1.000 | 0.743 | 3 | 0.539 | 1.000 | 0.956 | 3.32 | 3.25 | 0.001 | -12 | -22 | 36 |
|  |  | 1.000 | 0.743 | 3 | 0.539 | 1.000 | 0.956 | 3.32 | 3.25 | 0.001 | -22 | 14 | 2 |
|  |  | 1.000 | 0.743 | 4 | 0.473 | 1.000 | 0.956 | 3.31 | 3.24 | 0.001 | 12 | 68 | 6 |
|  |  | 1.000 | 0.743 | 7 | 0.338 | 1.000 | 0.956 | 3.31 | 3.24 | 0.001 | 8 | -64 | 60 |
|  |  | 1.000 | 0.743 | 6 | 0.375 | 1.000 | 0.956 | 3.30 | 3.24 | 0.001 | 52 | 24 | 2 |
|  |  | 1.000 | 0.743 | 1 | 0.743 | 1.000 | 0.956 | 3.30 | 3.23 | 0.001 | 28 | -60 | 30 |
|  |  | 1.000 | 0.743 | 2 | 0.624 | 1.000 | 0.956 | 3.29 | 3.22 | 0.001 | -8 | -48 | 54 |
|  |  | 1.000 | 0.743 | 2 | 0.624 | 1.000 | 0.956 | 3.29 | 3.22 | 0.001 | 44 | -4 | 52 |
|  |  | 1.000 | 0.743 | 2 | 0.624 | 1.000 | 0.956 | 3.28 | 3.21 | 0.001 | -34 | -8 | 12 |
|  |  | 1.000 | 0.743 | 1 | 0.743 | 1.000 | 0.956 | 3.27 | 3.21 | 0.001 | -40 | 14 | -34 |
|  |  | 1.000 | 0.743 | 2 | 0.624 | 1.000 | 0.956 | 3.27 | 3.20 | 0.001 | 40 | -74 | -8 |
|  |  | 1.000 | 0.743 | 3 | 0.539 | 1.000 | 0.956 | 3.27 | 3.20 | 0.001 | 26 | 14 | 46 |
|  |  | 1.000 | 0.743 | 2 | 0.624 | 1.000 | 0.956 | 3.26 | 3.19 | 0.001 | 4 | 50 | 28 |
|  |  | 1.000 | 0.743 | 1 | 0.743 | 1.000 | 0.956 | 3.25 | 3.19 | 0.001 | 4 | 12 | 40 |
|  |  | 1.000 | 0.743 | 1 | 0.743 | 1.000 | 0.956 | 3.25 | 3.19 | 0.001 | 18 | -26 | 54 |
|  |  | 1.000 | 0.743 | 1 | 0.743 | 1.000 | 0.956 | 3.24 | 3.18 | 0.001 | -14 | -8 | 52 |
|  |  | 1.000 | 0.743 | 2 | 0.624 | 1.000 | 0.956 | 3.24 | 3.18 | 0.001 | 50 | 18 | 34 |
|  |  | 1.000 | 0.743 | 2 | 0.624 | 1.000 | 0.956 | 3.24 | 3.18 | 0.001 | 48 | 12 | 50 |
|  |  | 1.000 | 0.743 | 1 | 0.743 | 1.000 | 0.956 | 3.23 | 3.17 | 0.001 | 34 | -54 | 64 |
|  |  | 1.000 | 0.743 | 1 | 0.743 | 1.000 | 0.956 | 3.22 | 3.16 | 0.001 | 44 | 20 | 24 |
|  |  | 1.000 | 0.743 | 2 | 0.624 | 1.000 | 0.956 | 3.21 | 3.15 | 0.001 | 14 | 58 | 26 |
|  |  | 1.000 | 0.743 | 1 | 0.743 | 1.000 | 0.956 | 3.21 | 3.15 | 0.001 | -56 | -54 | 32 |
|  |  | 1.000 | 0.743 | 1 | 0.743 | 1.000 | 0.956 | 3.21 | 3.15 | 0.001 | 0 | 62 | 34 |
|  |  | 1.000 | 0.743 | 1 | 0.743 | 1.000 | 0.956 | 3.20 | 3.14 | 0.001 | 34 | -52 | 50 |
|  |  | 1.000 | 0.743 | 1 | 0.743 | 1.000 | 0.980 | 3.19 | 3.13 | 0.001 | 70 | -26 | -12 |
|  |  | 1.000 | 0.743 | 1 | 0.743 | 1.000 | 0.983 | 3.18 | 3.12 | 0.001 | 30 | 30 | -18 |
|  |  | 1.000 | 0.743 | 1 | 0.743 | 1.000 | 0.983 | 3.17 | 3.11 | 0.001 | 50 | -18 | 2 |
|  |  | 1.000 | 0.743 | 1 | 0.743 | 1.000 | 0.983 | 3.16 | 3.10 | 0.001 | -34 | 10 | -34 |
|  |  | 1.000 | 0.743 | 1 | 0.743 | 1.000 | 0.983 | 3.16 | 3.10 | 0.001 | -24 | 64 | -2 |
|  |  | 1.000 | 0.743 | 1 | 0.743 | 1.000 | 0.983 | 3.16 | 3.10 | 0.001 | -58 | -48 | 36 |
| **UPPS-P - Sensation Seeking - Positive Contrast (NO IQ)** | | | | | | | | | | | | | |
| **Set-Level** | | **Cluster-Level** | | | | **Peak-Level** | | | | | **mm Coordinates** | | |
| *p* | *c* | p(FWE-corr) | p(FDR-corr) | equivk | p(unc) | p(FWE-corr) | p(FDR-corr) | *T* | equivZ | p(unc) | *x* | *y* | *z* |
| **0.033** | 37 | 0.651 | 0.293 | 36 | 0.040 | 0.298 | 0.392 | 4.62 | 4.45 | 0.000 | 18 | 6 | 62 |
|  |  | **0.004** | **0.006** | **159** | **0.000** | **0.358** | **0.392** | **4.55** | **4.39** | **0.000** | **48** | **42** | **-6** |
|  |  | 0.019 | 0.013 | 121 | 0.001 | 0.509 | 0.419 | 4.42 | 4.27 | 0.000 | 48 | 14 | 50 |
|  |  |  |  |  |  | 0.998 | 0.935 | 3.72 | 3.63 | 0.000 | 40 | 10 | 42 |
|  |  |  |  |  |  | 1.000 | 0.935 | 3.58 | 3.49 | 0.000 | 34 | 2 | 38 |
|  |  | 0.782 | 0.353 | 30 | 0.057 | 0.989 | 0.935 | 3.84 | 3.73 | 0.000 | 38 | 56 | 4 |
|  |  | 0.237 | 0.125 | 61 | 0.010 | 0.995 | 0.935 | 3.78 | 3.68 | 0.000 | 46 | -56 | 38 |
|  |  |  |  |  |  | 0.998 | 0.935 | 3.72 | 3.63 | 0.000 | 52 | -56 | 32 |
|  |  | 0.544 | 0.273 | 41 | 0.030 | 0.996 | 0.935 | 3.77 | 3.67 | 0.000 | 0 | 56 | 34 |
|  |  |  |  |  |  | 1.000 | 0.935 | 3.53 | 3.45 | 0.000 | 10 | 58 | 38 |
|  |  | 0.911 | 0.463 | 23 | 0.091 | 0.998 | 0.935 | 3.71 | 3.62 | 0.000 | -52 | 44 | 4 |
|  |  | 0.950 | 0.463 | 20 | 0.113 | 0.999 | 0.935 | 3.65 | 3.56 | 0.000 | -54 | -58 | 46 |
|  |  | 0.950 | 0.463 | 20 | 0.113 | 1.000 | 0.935 | 3.64 | 3.55 | 0.000 | 56 | 18 | 8 |
|  |  |  |  |  |  | 1.000 | 0.935 | 3.42 | 3.35 | 0.000 | 54 | 16 | 18 |
|  |  | 1.000 | 0.744 | 8 | 0.306 | 1.000 | 0.935 | 3.62 | 3.53 | 0.000 | 8 | 2 | 42 |
|  |  | 0.998 | 0.744 | 11 | 0.231 | 1.000 | 0.935 | 3.55 | 3.47 | 0.000 | 14 | 38 | 50 |
|  |  | 1.000 | 0.744 | 7 | 0.338 | 1.000 | 0.935 | 3.53 | 3.45 | 0.000 | 70 | -26 | -12 |
|  |  | 1.000 | 0.744 | 4 | 0.474 | 1.000 | 0.935 | 3.50 | 3.42 | 0.000 | -18 | -24 | 40 |
|  |  | 1.000 | 0.744 | 4 | 0.474 | 1.000 | 0.935 | 3.49 | 3.41 | 0.000 | -30 | 14 | 32 |
|  |  | 1.000 | 0.744 | 5 | 0.421 | 1.000 | 0.935 | 3.48 | 3.40 | 0.000 | 42 | -44 | -14 |
|  |  | 1.000 | 0.744 | 5 | 0.421 | 1.000 | 0.935 | 3.40 | 3.33 | 0.000 | 54 | -42 | 36 |
|  |  | 1.000 | 0.744 | 3 | 0.540 | 1.000 | 0.935 | 3.40 | 3.33 | 0.000 | 40 | 22 | 14 |
|  |  | 1.000 | 0.744 | 5 | 0.421 | 1.000 | 0.935 | 3.40 | 3.32 | 0.000 | -52 | 20 | 38 |
|  |  | 0.999 | 0.744 | 9 | 0.278 | 1.000 | 0.935 | 3.38 | 3.31 | 0.000 | 0 | 18 | 56 |
|  |  | 1.000 | 0.744 | 3 | 0.540 | 1.000 | 0.935 | 3.38 | 3.31 | 0.000 | 32 | -10 | 34 |
|  |  | 1.000 | 0.744 | 2 | 0.625 | 1.000 | 0.941 | 3.35 | 3.28 | 0.001 | -48 | -52 | 28 |
|  |  | 1.000 | 0.744 | 2 | 0.625 | 1.000 | 0.941 | 3.33 | 3.26 | 0.001 | 10 | -22 | 68 |
|  |  | 1.000 | 0.744 | 3 | 0.540 | 1.000 | 0.941 | 3.33 | 3.26 | 0.001 | -16 | -72 | 10 |
|  |  | 1.000 | 0.744 | 4 | 0.474 | 1.000 | 0.941 | 3.27 | 3.20 | 0.001 | -38 | -76 | -6 |
|  |  | 1.000 | 0.744 | 1 | 0.744 | 1.000 | 0.941 | 3.25 | 3.19 | 0.001 | -36 | -54 | -18 |
|  |  | 1.000 | 0.744 | 2 | 0.625 | 1.000 | 0.941 | 3.24 | 3.18 | 0.001 | 10 | -58 | 38 |
|  |  | 1.000 | 0.744 | 1 | 0.744 | 1.000 | 0.941 | 3.24 | 3.17 | 0.001 | 50 | 6 | 6 |
|  |  | 1.000 | 0.744 | 1 | 0.744 | 1.000 | 0.941 | 3.23 | 3.17 | 0.001 | -32 | 22 | 14 |
|  |  | 1.000 | 0.744 | 1 | 0.744 | 1.000 | 0.941 | 3.23 | 3.17 | 0.001 | 36 | 24 | 52 |
|  |  | 1.000 | 0.744 | 1 | 0.744 | 1.000 | 0.941 | 3.23 | 3.16 | 0.001 | -36 | 30 | -8 |
|  |  | 1.000 | 0.744 | 1 | 0.744 | 1.000 | 0.941 | 3.22 | 3.16 | 0.001 | 62 | -6 | -30 |
|  |  | 1.000 | 0.744 | 1 | 0.744 | 1.000 | 0.941 | 3.21 | 3.15 | 0.001 | 16 | -68 | 0 |
|  |  | 1.000 | 0.744 | 1 | 0.744 | 1.000 | 0.941 | 3.21 | 3.15 | 0.001 | -4 | 40 | 42 |
|  |  | 1.000 | 0.744 | 1 | 0.744 | 1.000 | 0.941 | 3.20 | 3.14 | 0.001 | 12 | 0 | 56 |
|  |  | 1.000 | 0.744 | 1 | 0.744 | 1.000 | 0.941 | 3.20 | 3.13 | 0.001 | 22 | -78 | 50 |
|  |  | 1.000 | 0.744 | 2 | 0.625 | 1.000 | 0.944 | 3.18 | 3.12 | 0.001 | 2 | -30 | -42 |
|  |  | 1.000 | 0.744 | 1 | 0.744 | 1.000 | 0.944 | 3.17 | 3.11 | 0.001 | 16 | 60 | 30 |

### Figure 2. BAS Fun Seeking Whole-brain Regression

Thresholded results from whole-brain regression of BAS Fun Seeking in the discovery sample (*N* = 143). The FWE-Bonferroni significant cluster in the pre-SMA is highlighted in green.


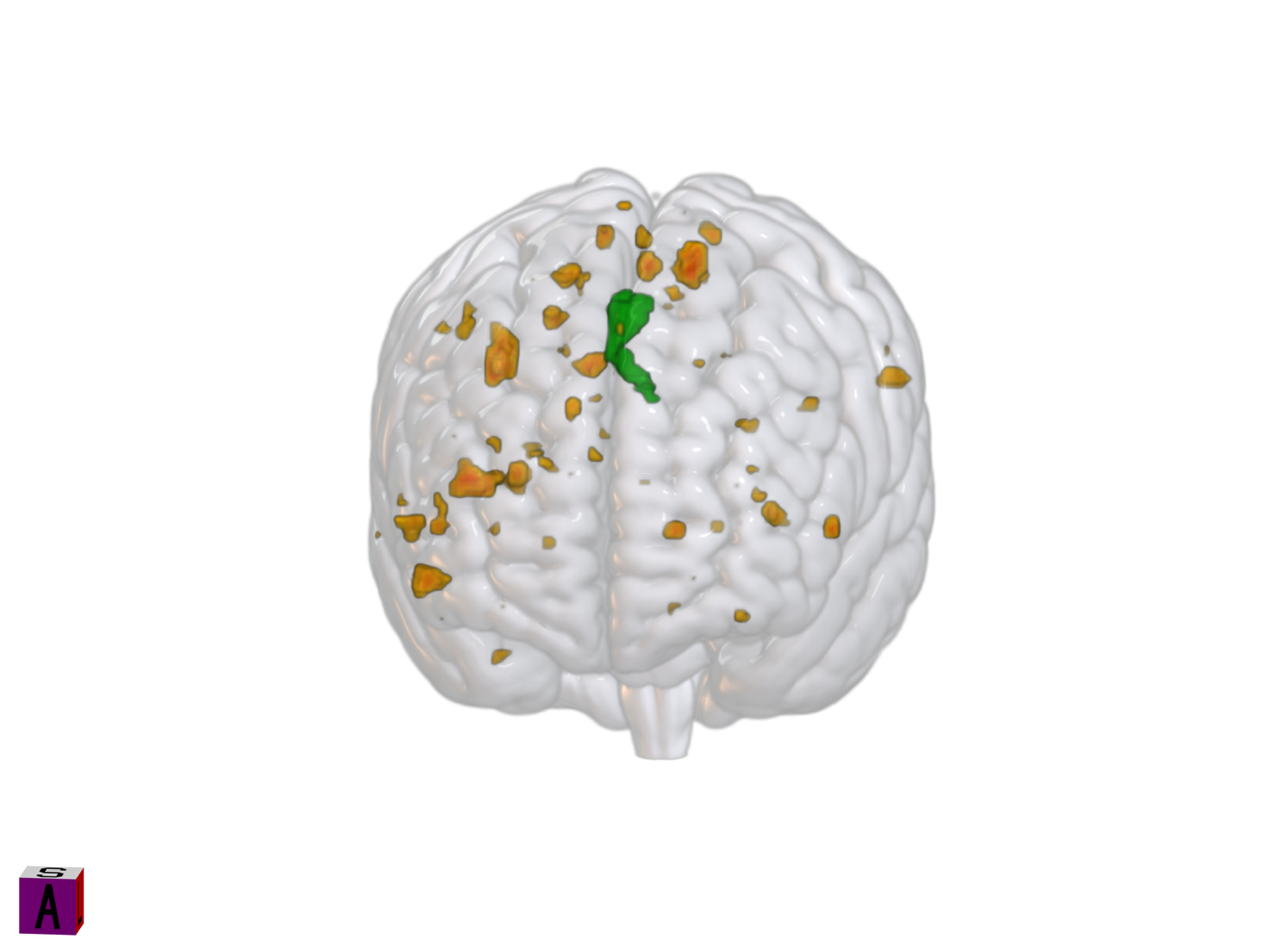


### Figure 3. UPPS-P Sensation Seeking Whole-brain Regression

Thresholded results from whole-brain regression of UPPS-P Sensation Seeking in the discovery sample (*N* = 143). The FWE-Bonferroni significant cluster in the R-vlPFC is highlighted in green.


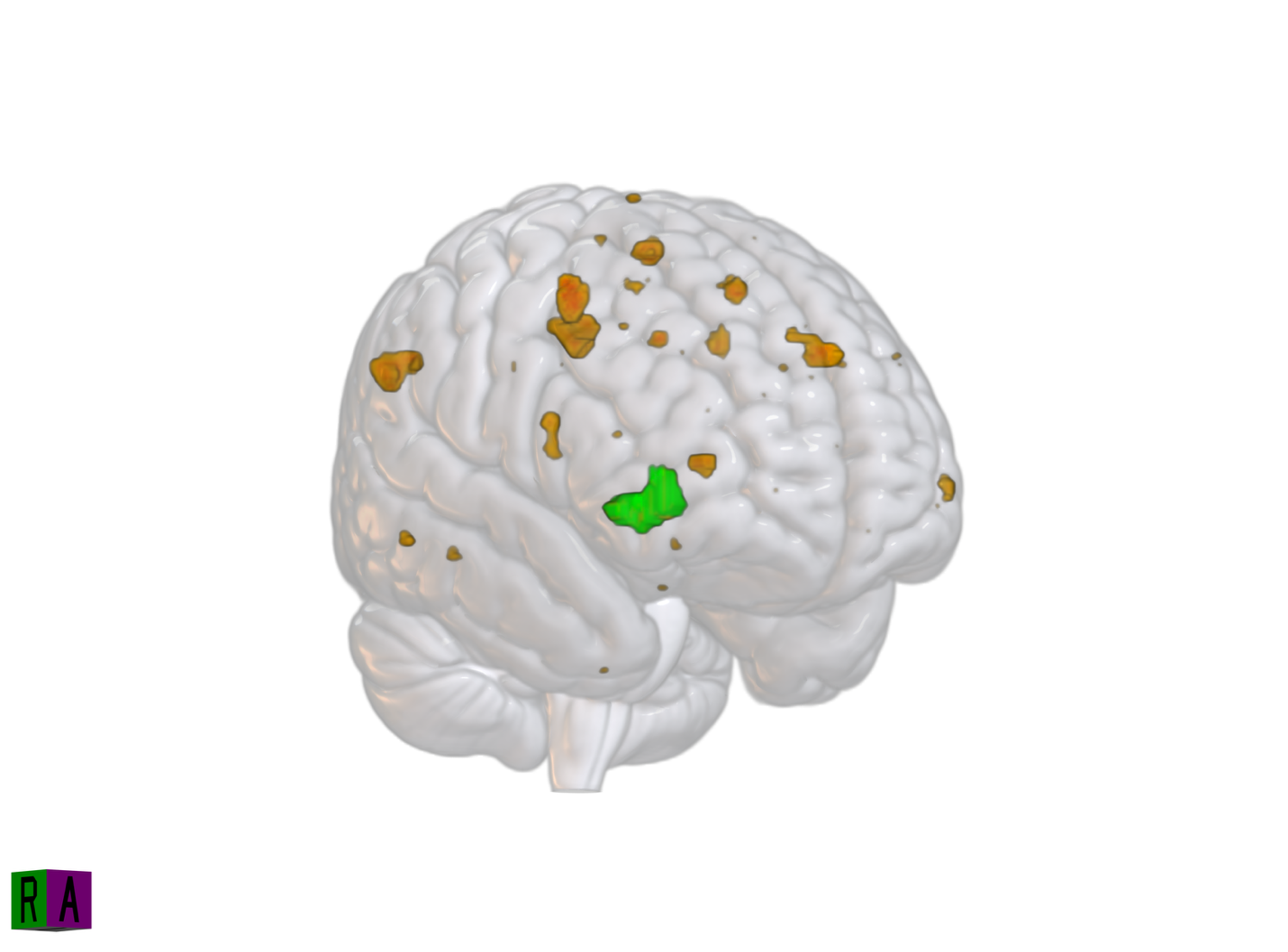


# Linear Regression Models of Pre-SMA Activity during RE and BAS Fun Seeking

| **Predictor** | **Estimate (*β*)** | **SE** | ***t*** | ***p*** | **Significance** |
| --- | --- | --- | --- | --- | --- |
| **Discovery Sample (*N* = 142)** |  |  |  |  |  |
| **Intercept** | 17.909 | 3.21 | 5.58 | <0.001 | *** |
| **Pre-SMA Activity during RE** | 2.729 | 0.508 | 5.372 | <0.001 | *** |
| **Sex** | 0.54 | 0.391 | 1.38 | 0.17 |  |
| **Age** | -0.124 | 0.055 | -2.254 | 0.026 | * |
| **NART IQ** | -0.033 | 0.025 | -1.319 | 0.189 |  |
| **HRSD** | 0.028 | 0.047 | 0.583 | 0.561 |  |
|  |  |  |  |  |  |
| **Model Fit** |  |  |  |  |  |
| **R² / Adjusted R²** | 0.215 / 0.186 |  |  |  |  |
| **F(df = 5, 136)** | 7.453 |  |  | <0.001 |  |
|  |  |  |  |  |  |
| **Replication Sample (*N* = 122)** |  |  |  |  |  |
| **Intercept** | 15.774 | 3.682 | 4.284 | <0.001 | *** |
| **Pre-SMA Activity during RE** | 0.883 | 0.416 | 2.125 | 0.036 | * |
| **Sex** | -0.649 | 0.491 | -1.323 | 0.189 |  |
| **Age** | 0.126 | 0.101 | 1.245 | 0.216 |  |
| **NART IQ** | -0.046 | 0.028 | -1.626 | 0.107 |  |
| **HRSD** | -0.107 | 0.034 | -3.123 | 0.002 | ** |
|  |  |  |  |  |  |
| **Model Fit** |  |  |  |  |  |
| **R² / Adjusted R²** | 0.135 / 0.097 |  |  |  |  |
| **F(df = 5, 116)** | 3.612 |  |  | 0.005 |  |
|  |  |  |  |  |  |
| **Combined Sample (*N* = 264)** |  |  |  |  |  |
| **Intercept** | 17.751 | 2.43 | 7.306 | <0.001 | *** |
| **Pre-SMA Activity during RE** | 1.486 | 0.328 | 4.536 | <0.001 | *** |
| **Sex** | 0.143 | 0.315 | 0.453 | 0.651 |  |
| **Age** | -0.071 | 0.049 | -1.466 | 0.144 |  |
| **NART IQ** | -0.039 | 0.019 | -2.071 | 0.039 | * |
| **HRSD** | -0.025 | 0.018 | -1.391 | 0.165 |  |
|  |  |  |  |  |  |
| **Model Fit** |  |  |  |  |  |
| **R² / Adjusted R²** | 0.097 / 0.079 |  |  |  |  |
| **F(df = 5, 258)** | 5.516 |  |  | <0.001 |  |

# Linear Regression Models of R-vlPFC Activity during RE and UPPS-P Sensation Seeking

| **Predictor** | **Estimate (*β*)** | **SE** | ***t*** | ***p*** | **Significance** |
| --- | --- | --- | --- | --- | --- |
| **Discovery Sample (*N* = 142)** |  |  |  |  |  |
| **Intercept** | 5.622 | 0.917 | 6.132 | <0.001 | *** |
| **R-vlPFC Activity during RE** | 0.408 | 0.091 | 4.495 | <0.001 | *** |
| **Sex** | 0.42 | 0.112 | 3.758 | <0.001 | *** |
| **Age** | -0.051 | 0.016 | -3.266 | 0.001 | ** |
| **NART IQ** | -0.016 | 0.007 | -2.265 | 0.025 | * |
| **HRSD** | 0.005 | 0.014 | 0.403 | 0.687 |  |
|  |  |  |  |  |  |
| **Model Fit** |  |  |  |  |  |
| **R² / Adjusted R²** | 0.234 / 0.206 |  |  |  |  |
| **F(df = 5, 136)** | 8.3 |  |  | <0.001 |  |
|  |  |  |  |  |  |
| **Replication Sample (*N* = 122)** |  |  |  |  |  |
| **Intercept** | 3.649 | 0.967 | 3.772 | <0.001 | *** |
| **R-vlPFC Activity during RE** | 0.016 | 0.086 | 0.19 | 0.849 |  |
| **Sex** | 0.133 | 0.129 | 1.028 | 0.306 |  |
| **Age** | 0.004 | 0.027 | 0.164 | 0.87 |  |
| **NART IQ** | -0.006 | 0.007 | -0.77 | 0.443 |  |
| **HRSD** | -0.031 | 0.009 | -3.504 | <0.001 | *** |
|  |  |  |  |  |  |
| **Model Fit** |  |  |  |  |  |
| **R² / Adjusted R²** | 0.121 / 0.083 |  |  |  |  |
| **F(df = 5, 116)** | 3.183 |  |  | 0.01 |  |

# Post Hoc Power Calculations

Sensitivity power analyses were conducted using the pwr package^16^ in R (version 4.4) to determine the minimal detectable effect sizes for all primary models (two-tailed *α* = 0.05, 80% power). Across samples, the design provided adequate sensitivity to detect small–to–moderate brain–behavior effects. For multiple linear regression models, effect size was indexed using Cohen’s *f*². In the discovery sample (*N* = 142), regression models examining associations between pre-supplementary motor area (pre-SMA) activity and BAS Fun Seeking, controlling for age, sex, and IQ, were powered to detect effects of f² = 0.057 (partial *r* = 0.23). In the replication sample (*N* = 122), minimal detectable effects ranged from *f*² = 0.067 to 0.068 (partial *r* = 0.25). In the combined sample (*N* = 264), which increased statistical precision, minimal detectable effects were *f*² = 0.030–0.031 (partial *r* = 0.17). For analysis of covariance (ANCOVA) group models, omnibus sensitivity was *f*² = 0.043 (partial *η*² = 0.041) for the four-group model and *f*² = 0.0408 (partial *η*² = 0.039) for the five-group model, controlling for age, sex, and IQ. The observed pre-SMA×HRSD interaction in the pooled regression model (F(1, 256) = 4.41) corresponded to partial *r* = 0.13 (*f*² = 0.017), within the range of reproducible effect sizes reported in large-scale individual-differences neuroimaging studies.

# Post Hoc Sensitivity Analysis

Psychotropic medication use was present in 7 of 264 participants (2.7%) in the pooled sample, including the antidepressants bupropion, citalopram, and mirtazapine, as well as the mood stabilizers lithium carbonate and lamotrigine. Sensitivity analyses were conducted to confirm that medication use did not account for the observed associations. First, primary regression models were re-estimated after excluding all medicated participants; results were unchanged, with comparable estimates for both the pre-SMA main effect on Fun Seeking (*β* = 1.52; SE = 0.33; *p* < 0.001) and the pre-SMA × HRSD interaction (*β* = −0.083; SE = 0.040; *p* = 0.039). Second, models including a binary covariate indexing any psychotropic use yielded nearly identical results (pre-SMA main effect: *β* = 1.47; SE = 0.33; *p* < 0.001; pre-SMA × HRSD interaction: *β* = −0.085; SE = 0.040; *p* = 0.035). Together, these analyses indicate that psychotropic medication use did not materially influence the reported findings.

# Exploratory Analysis in BD Sample

To contextualize impulsivity-related neural activity during RE within the BD group (*N* = 37), exploratory analyses were conducted. First, to evaluate the potential influence of ongoing pharmacological treatment on neural activity, a linear regression model was fitted with extracted beta values from the pre-SMA cluster as the dependent variable. Four classes of psychotropic medications were included as binary predictors (yes/no): antidepressants, benzodiazepines, mood stabilizers, and antipsychotics. Age, biological sex, and IQ were included as covariates. Then, the model was then refitted using a total medication load variable, reflecting cumulative psychotropic burden across medication classes, to assess whether overall medication exposure influenced pre-SMA activity.

Then, additional models were fitted to test whether illness characteristics influenced pre-SMA activity during RE. In one model, the number of prior manic/hypomanic episodes was included as a predictor alongside age, sex, and IQ. In a second model, illness duration was calculated by subtracting age of BD onset from age at MRI, and this variable was used to predict pre-SMA activity, adjusting for the same covariates. These exploratory models aimed to assess whether cumulative illness burden or chronicity impacted impulsivity-related neural activity during RE. Finally, a linear model was estimated using BDI vs BDII as a binary covariate to ensure that observed differences were not due to BD subtype.

## Medication Types as Predictors of Pre-SMA Activity in BD Sample

| **Predictor** | **Estimate (*β*)** | **SE** | ***t*** | ***p*** | **Significance** |
| --- | --- | --- | --- | --- | --- |
| **BD Sample (*N* = 37)** | | | | | |
| **Intercept** | 2.369 | 1.555 | 1.524 | 0.138 |  |
| **Sex** | -0.068 | 0.197 | -0.343 | 0.734 |  |
| **Age** | 0.005 | 0.019 | 0.269 | 0.79 |  |
| **NART IQ** | -0.021 | 0.013 | -1.572 | 0.127 |  |
| **Antidepressants** | -0.205 | 0.154 | -1.324 | 0.196 |  |
| **Benzodiazepines** | -0.874 | 0.327 | -2.672 | 0.012 | * |
| **Mood Stabilizers** | -0.289 | 0.161 | -1.799 | 0.083 |  |
| **Antipsychotics** | 0.06 | 0.165 | 0.362 | 0.72 |  |
| **Model Fit** |  |  |  |  |  |
| **R² / Adjusted R²** | 0.374 / 0.223 |  |  |  |  |
| **F(df = )** | 2.476 (7, 29) |  |  | 0.04 |  |

**Multicollinearity diagnostics were conducted using variance inflation factors (VIF) computed with the car package in R.*^17^ *All predictors demonstrated acceptable tolerance (VIF range: 1.11–1.29).*

## Medication Load (Total) as a Predictor of Pre-SMA Activity in BD Sample

| **Predictor** | **Estimate (*β*)** | **SE** | ***t*** | ***p*** | **Significance** |
| --- | --- | --- | --- | --- | --- |
| **BD Sample (*N* = 37)** | | | | | |
| Intercept | 2.864 | 1.751 | 1.636 | 0.112 |  |
| Sex | 0.086 | 0.199 | 0.43 | 0.67 |  |
| Age | −0.001 | 0.021 | −0.038 | 0.97 |  |
| NART IQ | −0.025 | 0.015 | −1.750 | 0.09 |  |
| Medication Load (Total) | −0.055 | 0.047 | −1.167 | 0.252 |  |
| **Model Fit** |  |  |  |  |  |
| R² / Adjusted R² | 0.188 / 0.086 | |  |  |  |
| F(df = 4, 32) | 1.849 |  |  | 0.144 |  |

## Number of Manic/hypomanic Episodes as Predictors of Pre-SMA Activity in BD Sample

| **Predictor** | **Estimate (*β*)** | **SE** | ***t*** | ***p*** | **Significance** |
| --- | --- | --- | --- | --- | --- |
| **BD Sample (*N* = 37)** | | | | | |
| **Intercept** | 3.621 | 1.633 | 2.217 | 0.034 | * |
| **Sex** | 0.224 | 0.237 | 0.948 | 0.351 |  |
| **Age** | -0.011 | 0.02 | -0.531 | 0.599 |  |
| **NART IQ** | -0.031 | 0.014 | -2.251 | 0.032 | * |
| **Number of Manic Episodes** | -0.062 | 0.061 | -1.017 | 0.317 |  |
| **Number of Hypomanic Episodes** | -0.002 | 0.005 | -0.431 | 0.67 |  |
| **Model Fit** |  |  |  |  |  |
| **R² / Adjusted R²** | 0.184 / 0.052 |  |  |  |  |
| **F(df = )** | 1.397 (5, 31) |  |  | 0.253 |  |

## Duration of Illness as Predictors of Pre-SMA Activity in BD Sample

| **Predictor** | **Estimate (*β*)** | **SE** | ***t*** | ***p*** | **Significance** |
| --- | --- | --- | --- | --- | --- |
| **BD Sample (*N* = 37)** | | | | | |
| **Intercept** | 3.616 | 1.681 | 2.151 | 0.039 | * |
| **Sex** | 0.131 | 0.212 | 0.618 | 0.541 |  |
| **Age** | -0.007 | 0.025 | -0.28 | 0.781 |  |
| **NART IQ** | -0.032 | 0.014 | -2.324 | 0.027 | * |
| **Duration of BD Illness** | -0.005 | 0.022 | -0.228 | 0.821 |  |
| **Model Fit** |  |  |  |  |  |
| **R² / Adjusted R²** | 0.155 / 0.049 |  |  |  |  |
| **F(df = )** | 1.463 (4, 32) |  |  | 0.237 |  |

## Effect of BD I versus II as Predictors of Pre-SMA Activity in BD Sample

| **Predictor** | **Estimate (*β*)** | **SE** | ***t*** | ***p*** | **Significance** |
| --- | --- | --- | --- | --- | --- |
| **BD Sample (*N* = 37)** | | | | | |
| **Intercept** | 3.280 | 1.616 | 2.029 | 0.051 |  |
| **Sex** | 0.282 | 0.233 | 1.210 | 0.235 |  |
| **Age** | -0.009 | 0.020 | -0.463 | 0.647 |  |
| **NART IQ** | -0.033 | 0.013 | -2.466 | 0.020 | * |
| **BD I vs. II** | 0.249 | 0.188 | 1.326 | 0.194 |  |
| **Model Fit** |  |  |  |  |  |
| **R² / Adjusted R²** | 0.198 / 0.097 |  |  |  |  |
| **F(df = )** | 1.197 (4, 32) |  |  | 0.124 |  |

# Exploratory Analysis: Whole-brain Regression in Replication Sample

As exploratory analyses, whole-brain regressions were conducted for BAS Fun Seeking in the replication sample (*N* = 122) following the same methods described for Aim 1. This analysis identified a significant cluster in the left anterior prefrontal cortex (L-aPFC; Brodmann Area 10; *p*_FWE_ = 0.031, *k* = 144), as well as the precuneus (*p*_FWE_ = 0.010, *k* = 184; Figure 4). For these unique clusters identified in the replication sample, follow-up models with extracted beta values were used, as in Aim 2, to test associations among neural activity and Fun Seeking in each sample, and the impact of these association with affective or anxiety symptoms as covariates, as in Aim 3, to test potential moderating effects of these symptoms on neural activity-Fun Seeking associations.

### Figure 4. Significant clusters of neural activity associated with Fun Seeking in whole-brain regression in the replication sample.


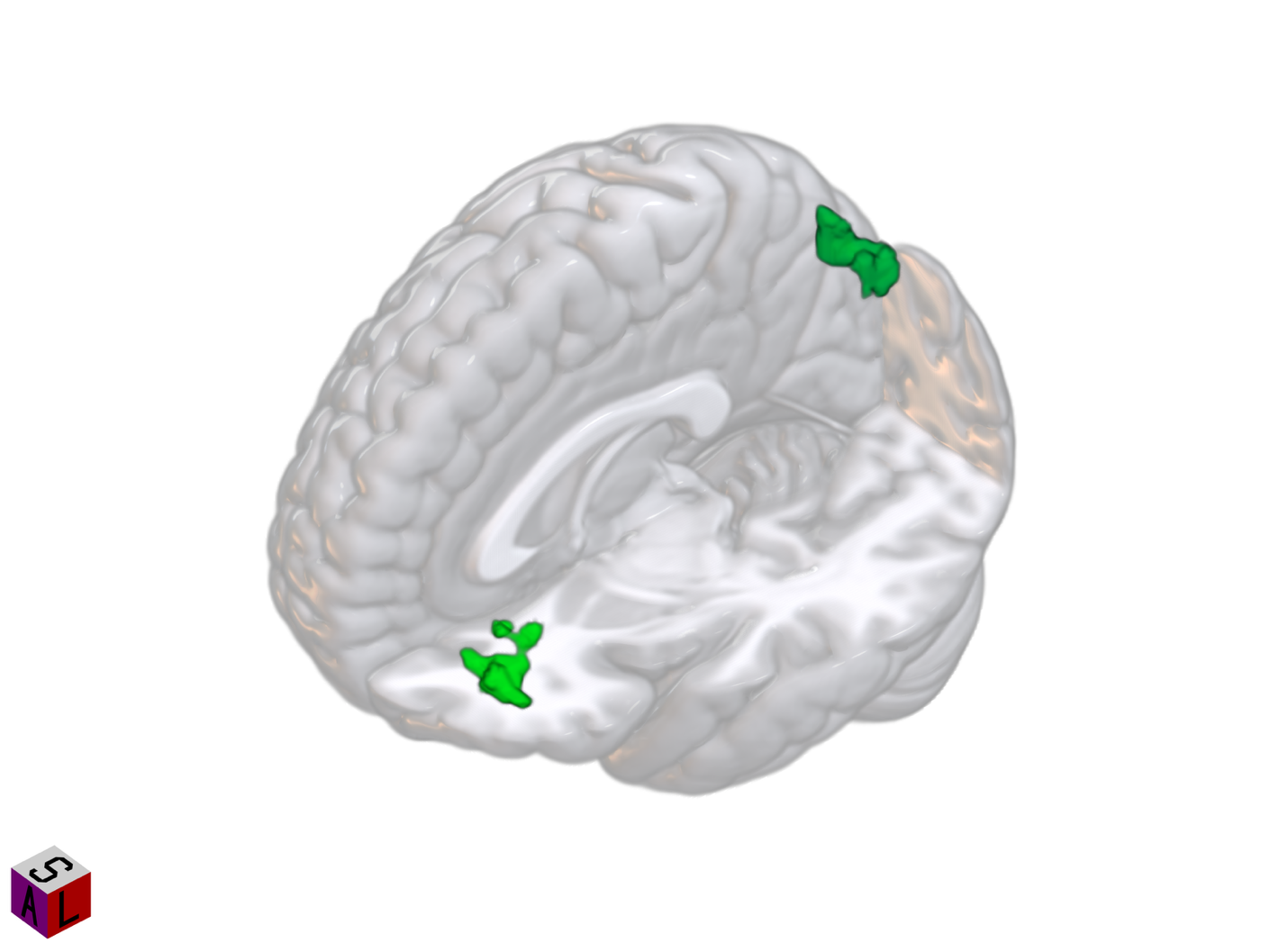


## Linear Models for the Left Anterior Prefrontal Cortex and Fun Seeking

To evaluate whether the left anterior prefrontal cortex (L-aPFC) cluster identified in the exploratory whole-brain analyses was associated with approach-related impulsivity, linear regression models were conducted using extracted beta values of RE-related activity. Fun Seeking was entered as the dependent variable, and models were conducted in the discovery and replication samples separately, controlling for age, sex, and IQ. In the replication sample, greater L-aPFC activity significantly predicted higher Fun Seeking scores both without affective or anxiety symptom severity covariates (*β* = 2.48, *p* < 0.001) and after adjusting for depressive symptom severity (HRSD; *β* = 2.37, *p* < 0.001), anxiety symptom severity (HAMA; *β* = 2.45, *p* < 0.001), or manic/hypomanic symptom severity (YMRS; *β* = 2.47, *p* < 0.001). In contrast, in the discovery sample, L-aPFC activity was not significantly associated with Fun Seeking in any model, either without affective or anxiety covariates (*β* = 0.31, *p* = 0.666) or when controlling for HRSD (*β* = 0.37, *p* = 0.607), HAMA (*β* = 0.39, *p* = 0.594), or YMRS (*β* = 0.47, *p* = 0.510). These findings indicate that, although L-aPFC activity was associated with Fun Seeking in the replication sample, this effect did not replicate in the discovery sample and therefore did not meet criteria for a reproducible neural correlate of Fun Seeking.

## Linear Models for the Precuneus and Fun Seeking

RE–related activity in the precuneus cluster, identified in the exploratory whole-brain regression, was then tested for associations with Fun Seeking. Linear regression models were conducted in the discovery and replication samples using extracted beta values from the precuneus cluster. In contrast to the L-aPFC findings, precuneus activity during RE showed a consistent positive association with Fun Seeking across both samples and across all affective and anxiety symptom severity covariate models. In the discovery sample, greater precuneus activity showed a trend-level association with higher Fun Seeking in the model without affective or anxiety covariates (*β* = 0.71, *p* = 0.063) and was significantly associated with higher Fun Seeking when adjusting for depressive symptom severity (HRSD; *β* = 0.78, *p* = 0.042), anxiety symptom severity (HAMA; *β* = 0.84, *p* = 0.031), or manic/hypomanic symptom severity (YMRS; *β* = 0.86, *p* = 0.022). In the replication sample, greater precuneus activity significantly predicted higher Fun Seeking in all models: without covariates (*β* = 1.09, *p* < 0.001), and when adjusting for HRSD (*β* = 1.01, *p* < 0.001), HAMA (*β* = 1.03, *p* < 0.001), or YMRS (*β* = 1.09, *p* < 0.001). In the combined sample (*N* = 264), greater precuneus activity remained a significant positive predictor of Fun Seeking across all models, including those adjusting for depressive symptom severity (HRSD; *β* = 0.96, *p* = 0.008), anxiety symptom severity (HAMA; *β* = 0.96, *p* = 0.009), and manic/hypomanic symptom severity (YMRS; *β* = 0.98, *p* = 0.004), as well as in the model without affective or anxiety covariates (*β* = 0.97, *p* = 0.006), indicating that this association was robust in the combined sample.

## Supplemental Discussion

Unlike the L-aPFC findings, precuneus–Fun Seeking associations remained robust in both samples irrespective of depressive, anxiety, or manic/hypomanic symptom severity, indicating reproducibility of this association across samples and model specifications. This pattern is consistent with the subthreshold precuneus activation observed in the initial whole-brain regression of Fun Seeking in the discovery sample, which did not reach cluster-level significance (Figure 5). As the precuneus is a core hub of the default mode network implicated in self-referential cognition, autobiographical memory retrieval, and prospective simulation of future actions,^18^ its engagement during RE may reflect internally driven, self-relevant anticipation of potential reward outcomes, consistent with approach-related motivational tendencies indexed by Fun Seeking. Thus, precuneus activity during RE likely captures the self-referential, future-oriented component of approach motivation rather than the impulsivity-related action tendencies captured by pre-SMA activity. Moreover, precuneus–Fun Seeking associations were stable regardless of current affective or anxiety symptom severity, suggesting that this effect reflects a trait-like self-referential, future-oriented component of approach motivation rather than an affective or anxiety-driven processes.

### Figure 5. An overlay of the discovery sample whole-brain regression results (yellow) on top of the replication sample whole-brain regression results (green), showing overlapping activity in the precuneus.


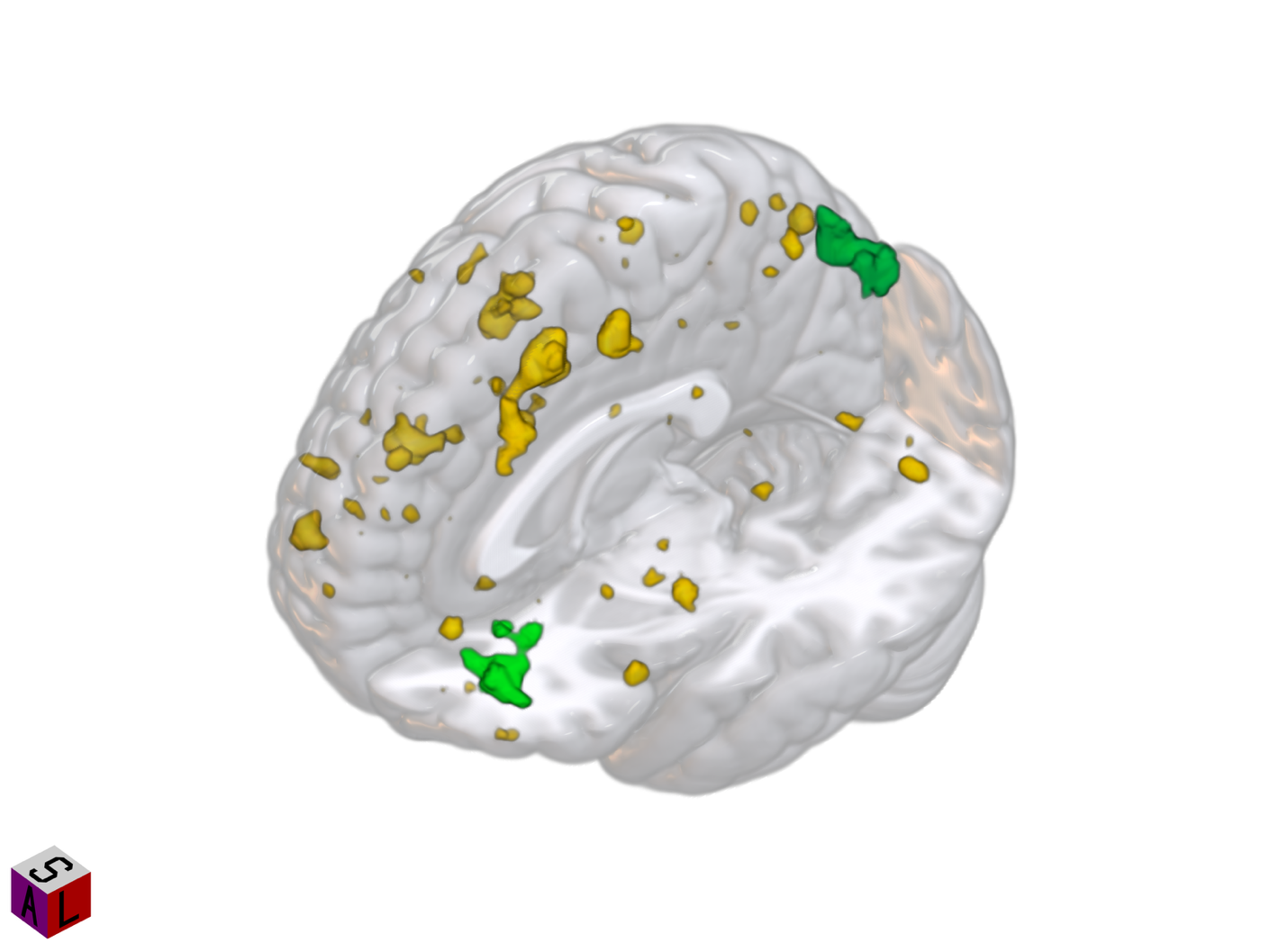


##

# References

1. First M, Williams J, Karg R, Spitzer RL. Structured Clinical Interview for DSM-5—Research Version (SCID-5 for DSM-5, Research Version; SCID-5-RV). *Am Psychiatr Assoc*. Published online 2015.

2. Hamilton M. A RATING SCALE FOR DEPRESSION. *J Neurol Neurosurg Psychiatry*. 1960;23(1):56-62. doi:10.1136/jnnp.23.1.56

3. Young RC, Biggs JT, Ziegler VE, Meyer DA. A Rating Scale for Mania: Reliability, Validity and Sensitivity. *Br J Psychiatry*. 1978;133(5):429-435. doi:10.1192/bjp.133.5.429

4. Kay SR, Fiszbein A, Opler LA. The Positive and Negative Syndrome Scale (PANSS) for Schizophrenia. *Schizophr Bull*. 1987;13(2):261-276. doi:10.1093/schbul/13.2.261

5. Brown HE, Hart KL, Snapper LA, Roffman JL, Perlis RH. Impairment in delay discounting in schizophrenia and schizoaffective disorder but not primary mood disorders. *Npj Schizophr*. 2018;4(1):9. doi:10.1038/s41537-018-0050-z

6. Folstein MF, Folstein SE, McHugh PR. Mini-mental state. *J Psychiatr Res*. 1975;12(3):189-198. doi:10.1016/0022-3956(75)90026-6

7. Blair JR, Spreen O. Predicting premorbid IQ: A revision of the national adult reading test. *Clin Neuropsychol*. 1989;3(2):129-136. doi:10.1080/13854048908403285

8. Carver CS, White TL. Behavioral inhibition, behavioral activation, and affective responses to impending reward and punishment: The BIS/BAS Scales. *J Pers Soc Psychol*. 1994;67(2):319-333. doi:10.1037/0022-3514.67.2.319

9. Maack DJ, Ebesutani C. A re‐examination of the BIS/BAS scales: Evidence for BIS and BAS as unidimensional scales. *Int J Methods Psychiatr Res*. 2018;27(2):e1612. doi:10.1002/mpr.1612

10. Gray JA. The psychophysiological basis of introversion-extraversion. *Behav Res Ther*. 1970;8(3):249-266. doi:10.1016/0005-7967(70)90069-0

11. Whiteside SP, Lynam DR. The Five Factor Model and impulsivity: using a structural model of personality to understand impulsivity. *Personal Individ Differ*. 2001;30(4):669-689. doi:10.1016/S0191-8869(00)00064-7

12. Cyders MA, Smith GT, Spillane NS, Fischer S, Annus AM, Peterson C. Integration of impulsivity and positive mood to predict risky behavior: Development and validation of a measure of positive urgency. *Psychol Assess*. 2007;19(1):107-118. doi:10.1037/1040-3590.19.1.107

13. Hamilton M. THE ASSESSMENT OF ANXIETY STATES BY RATING. *Br J Med Psychol*. 1959;32(1):50-55. doi:10.1111/j.2044-8341.1959.tb00467.x

14. Chase HW, Fournier JC, Bertocci MA, et al. A pathway linking reward circuitry, impulsive sensation-seeking and risky decision-making in young adults: identifying neural markers for new interventions. *Transl Psychiatry*. 2017;7(4):e1096-e1096. doi:10.1038/tp.2017.60

15. Fortin JP. neuroCombat: Harmonization of multi-site imaging data with ComBat. Published online 2020. https://github.com/Jfortin1/neuroCombat_Rpackage

16. Champely S. pwr: Basic Functions for Power Analysis. Published online February 1, 2006:1.3-0. doi:10.32614/CRAN.package.pwr

17. Fox J, Weisberg S, Price B. car: Companion to Applied Regression. Published online May 1, 2001:3.1-3. doi:10.32614/CRAN.package.car

18. Cavanna AE, Trimble MR. The precuneus: a review of its functional anatomy and behavioural correlates. *Brain*. 2006;129(3):564-583. doi:10.1093/brain/awl004
